# The impact of mindfulness training on emotional resilience and job engagement among NICU and PICU Saudi and Egyptian nurses: a quasi experimental comparative study

**DOI:** 10.3389/fpsyt.2025.1701580

**Published:** 2026-02-19

**Authors:** Amal I. Khalil, Abeer Esawi, Ebtesam Abdulshakoor, Omayma M. Abu Samra

**Affiliations:** 1 King Abdullah International Medical Research Center, Jeddah, Saudi Arabia; 2 College of Nursing, King Saud bin Abdulaziz University for Health Sciences, Jeddah, Saudi Arabia; 3 Ministry of National Guard Health Affairs (MNGHA), Jeddah, Saudi Arabia; 4 Faculty of Nursing, Menoufia University, Shebeen El-Kom, Egypt; 5 Faculty of Nursing, Cairo University, Cairo, Egypt; 6 Faculty of Nursing, Mansoura University, Mansoura, Egypt

**Keywords:** mindfulness, emotional resilience, job engagement, NICU, PICU, nurses, Saudi Arabia, Egypt

## Abstract

**Background:**

Nurses working in Neonatal and Pediatric Intensive Care Units (NICU/PICU) experience high levels of stress that can diminish emotional resilience and job engagement. Mindfulness-based interventions (MBIs) are known to enhance psychological well-being; however, evidence in culturally diverse settings, such as Saudi Arabia and Egypt, remains limited.

**Objective:**

This study assessed the effects of mindfulness training on emotional resilience, job engagement, and mindfulness and compared the outcomes between NICU and PICU nurses in both countries.

**Methods:**

A quasi-experimental pre-post-comparative design was used. The participants were nurses from the King Abdullah Specialized Children’s Hospital (Saudi Arabia) and Mansoura University Pediatric Hospital (Egypt). Emotional resilience, engagement, and mindfulness were measured using validated tools, including the Five-Facet Mindfulness Questionnaire (FFMQ). An eight-session Mindfulness-Based Stress Reduction (MBSR) program was delivered in person, supported by video recordings to address scheduling constraints. Data were analyzed using paired t-tests, ANOVA, and Pearson’s correlation (p < 0.05).

**Results:**

The baseline demographics of the Egyptian and Saudi nurses were comparable. Post-intervention, both groups showed significant improvements in mindfulness, engagement, and resilience. Egyptian nurses demonstrated greater gains, with mindfulness increasing from 74.8 to 164.0, engagement from 24.2 to 81.3, and resilience from 13.1 to 36.3. Saudi nurses also improved, with mindfulness increasing from 79.7 to 136.5, engagement from 26.1 to 72.6, and resilience from 14.7 to 28.7. High levels across all domains were achieved by 91.7% of the Egyptian nurses and 25.0–73.3% of the Saudi nurses.

**Conclusion:**

Mindfulness training is an effective strategy for enhancing emotional resilience and job engagement among NICU and PICU nurses. Flexible delivery can reduce implementation barriers, supporting culturally adapted MBIs to improve nurses’ well-being.

## Introduction

Nurses working in Neonatal Intensive Care Units (NICUs) and Pediatric Intensive Care Units (PICUs) often operate in high-stress environments due to the critical nature of their work. This stress can lead to reduced emotional resilience and job engagement, affecting their well-being and the quality of patient care (1, 2). Emotional resilience is crucial for nurses to manage the emotional demands of their jobs and to maintain high levels of professional performance. Job engagement, the level of enthusiasm and connection nurses feel towards their work, is essential for ensuring sustained productivity and job satisfaction.

Mindfulness training has become a promising intervention for improving healthcare professionals’ emotional resilience and job engagement in recent years. Practicing mindfulness, which involves being aware of one’s thoughts, feelings, and surroundings moment by moment, can reduce stress and enhance mental well-being. Numerous studies have shown that mindfulness training has positive effects on decreasing burnout and enhancing emotional regulation among nurses (3, 4). Although there is a growing body of evidence supporting mindfulness interventions (5, 6), there is limited research on their impact on NICU and PICU nurses, especially in non-Western contexts such as Saudi Arabia and Egypt (7, 8). Given the cultural and occupational differences in these regions, it is crucial to investigate whether mindfulness training yields similar benefits for nurses in these settings. To address this gap, the current quasi-experimental study aimed to assess the effects of mindfulness training on emotional resilience and job engagement among NICU and PICU nurses in Saudi Arabia and Egypt (9, 10).

Despite the growing body of literature supporting the positive impact of mindfulness training on emotional resilience and job engagement (11, 12), there is a research gap in understanding the specific effects of mindfulness training on emotional resilience and job engagement among nurses in Neonatal Intensive Care Units (NICU)and in Pediatric Intensive Care (PICU) (13). This gap is particularly evident in non-Western contexts, such as Saudi Arabia and Egypt, where cultural and occupational differences may influence outcomes (13–15). This study aims to fill this research gap by conducting a quasi-experimental study to investigate the effects of mindfulness intervention on emotional resilience and job engagement among nurses working in neonatal and pediatric intensive care units in Saudi Arabia and Egypt, thereby contributing to the overall improvement of patient care and nurses’ well-being (14).

Previous studies have highlighted the significant impact of high stress and emotional exhaustion levels on the well-being and job engagement of nurses working in the NICU and PICU (16, 17). Mindfulness is defined as the practice of being fully present and engaged in the current moment, acknowledging and accepting one’s thoughts and feelings without judgment (18). Mindfulness training plays a crucial role in enhancing emotional resilience and job engagement among NICU and PICU nurses (19).

Emotional resilience is crucial for individuals working in high-stress environments, such as healthcare professionals in intensive care units, as it can help them maintain their well-being and job engagement despite the challenges. According to Southwick et al. (20), emotional resilience is the ability to adapt to emotional stress and recover quickly from emotional stressors. This definition emphasizes the importance of coping with emotional stress and recovering efficiently.

Job engagement among NICU nurses can be defined as the emotional and mental connection that nurses feel toward their work in the NICU, influencing their dedication to providing high-quality patient care and their sense of fulfillment and commitment to their roles. Job engagement refers to a positive, fulfilling, work-related state of mind characterized by vigor, dedication, and absorption (21). This definition highlights the aspects of energy, involvement, and absorption related to work, which are crucial components of job engagement among NICU nurses. Moreover, in these high-stress environments, nurses’ emotional well-being is directly linked to the quality of care provided to vulnerable patients. Research has shown that nurses with higher levels of emotional resilience and job engagement report lower burnout rates and higher patient satisfaction (22).

The demanding nature of their work requires nurses in these units to manage their emotions effectively, remain present, and respond to challenging situations with clarity and compassion. Mindfulness interventions have been shown to reduce stress levels, decrease burnout, and improve compassion satisfaction, ultimately leading to increased emotional resilience among nurses (23, 24). Furthermore, mindfulness facilitates work engagement by enhancing meaningfulness, emotion regulation, and job competence, thereby positively impacting overall job satisfaction and performance among healthcare professionals.

Cultural differences play a crucial role in the effectiveness of mindfulness interventions. Research suggests that perceptions and acceptance of mindfulness practices can vary widely across cultures, affecting both implementation and outcomes (23) In Saudi Arabia and Egypt, specific cultural factors and societal norms may shape how nurses engage with and respond to mindfulness training programs (24). Elements such as religious beliefs, traditional healing practices, and prevailing attitudes toward mental health and well-being can significantly influence the receptivity and impact of these interventions (25). Incorporating a cross-cultural perspective into this study allowed for the exploration of the complex relationships between mindfulness, emotional resilience, and job engagement within these distinct cultural contexts (26). This approach not only enhances our understanding of the benefits of mindfulness for nurses in high-stress environments but also guides the design of interventions that are culturally sensitive and aligned with local values and frameworks in the future (27).

## Significance of the study

This study fills a vital gap in the research on mindfulness training for nurses in high-stress settings, such as NICUs and PICUs, particularly in the Middle East. In Saudi Arabia, research in this area is developing but is restricted, with fewer than ten studies. Egypt has even fewer studies, with less than five notable ones (27, 28). This study aims to fill this gap by examining the impact of mindfulness training on emotional resilience and job engagement among nurses in these countries. Given that past research has suggested a clear link between nurses’ emotional well-being and patient satisfaction, albeit without conclusive evidence, it is essential to investigate this connection further. This can be achieved through mindfulness interventions designed to enhance the mental and emotional health of healthcare professionals, thereby improving the quality of patient care. Moreover, the potential benefits of this study include improved emotional resilience and job engagement among nurses, leading to better psychological well-being, increased job satisfaction, lower burnout rates, and greater retention. These improvements can enhance the quality of care provided to neonates and children, resulting in better patient outcomes, such as improved recovery rates and overall health. Thus, this study aims to enhance the professional lives of nurses and the health and well-being of vulnerable patients.

## Theoretical framework

**Figure 1:**
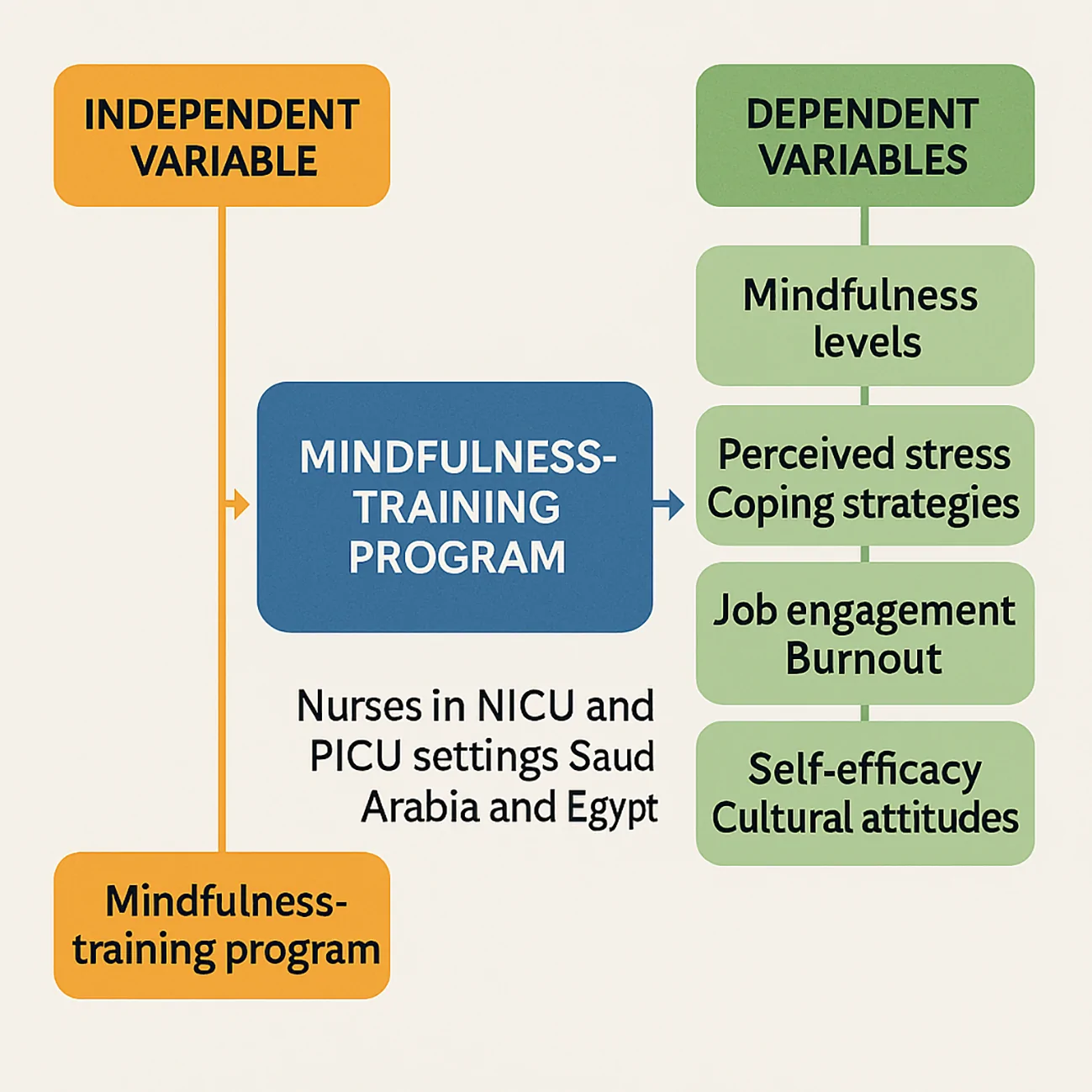
Visual image for the theoretical framework supporting the study variables (dependent and independent).

Thus, blending diverse theoretical frameworks to create a mindfulness training initiative is crucial. This initiative aims to bolster emotional resilience and work engagement among nurses in the NICU and PICU environments in Saudi Arabia and in Egypt. Rooted in mindfulness theory (29, 30), the core of this program lies in its structure, content, and delivery method. The focal point was the mindfulness training program as the independent variable, with changes in nurses’ mindfulness levels evaluated through rigorous self-reports or observational assessments covering elements such as present-moment awareness and self-compassion (**Figure 1**).

Furthermore, mindfulness training plays a crucial role as a coping mechanism for handling stress in demanding healthcare settings. Leveraging stress and coping theory (31, 32), variables such as perceived stress levels, coping strategies, and emotional regulation abilities were assessed using self-report scales examining perceived stress and coping styles.

Moreover, tapping into the Job Demands-Resources model (33, 34), mindfulness training is viewed as a job resource that aids nurses in managing their workload challenges. Changes in job engagement and burnout were measured using self-report scales that evaluated vigor at work and emotional exhaustion. Additionally, according to Social Cognitive Theory (35, 36), mindfulness training enhances nurses’ belief in effective stress management. Self-efficacy beliefs regarding stress management and job performance were appraised using specific self-report scales.

Blending diverse theoretical frameworks to create a mindfulness training initiative is thus crucial. This initiative aims to bolster emotional resilience and work engagement among nurses in the NICU and PICU environments in Saudi Arabia and Egypt. Rooted in mindfulness theory (37), the core of this program lies in its structure, content, and delivery method. The focal point was the mindfulness training program as the independent variable, with changes in nurses’ mindfulness levels evaluated through rigorous self-reports or observational assessments covering elements such as present-moment awareness and self-compassion (**Figure 1**).

Furthermore, mindfulness training plays a crucial role as a coping mechanism for handling stress in healthcare settings. Leveraging stress and coping theory (38), variables such as perceived stress levels, coping strategies, and emotional regulation abilities were assessed using self-report scales that examined perceived stress and coping styles.

Moreover, tapping into the Job Demands-Resources model (38), mindfulness training is viewed as a job resource that aids nurses in managing their workload. Changes in job engagement and burnout were measured using self-report scales that evaluated vigor and emotional exhaustion at work. Additionally, according to Social Cognitive Theory (35), mindfulness training enhances nurses’ belief in effective stress management. Self-efficacy beliefs regarding stress management and job performance were appraised using specific self-report scales.

Recognizing the importance of cultural nuances (36), this proposal includes a culturally tailored mindfulness training program. This program, designed to align with local values, is pivotal to our approach in this study. Cultural attitudes toward mindfulness changes are gauged through qualitative methods, such as focus groups or interviews, to capture the perceptions of cultural relevance. A comprehensive mindfulness training program was developed by cohesively integrating these theoretical foundations. This program offers thorough insights into its potential impact on emotional resilience and work engagement among nurses in the NICU and PICU settings in Saudi Arabia and Egypt, enriching our understanding of its potential benefits for nurses.

## Aim of the study

This quasi-experimental study aimed to investigate the impact of mindfulness training on emotional resilience and job engagement among nurses working in Neonatal Intensive Care Units (NICU) and Pediatric Intensive Care Units (PICU) in Saudi Arabia and Egypt.

### Study objectives, hypotheses, and research questions

#### Specific objectives

- Assess baseline and changes in emotional resilience, job engagement, and psychological outcomes (FFMQ) among NICU and PICU nurses in Saudi Arabia and Egypt.
- Develop and implement a mindfulness training program tailored for NICU and PICU nurses.
- Compare the effects of mindfulness training on emotional resilience, job engagement, and FFMQ outcomes between Saudi and Egyptian nurses.
- Examine the cultural factors influencing the effectiveness of mindfulness training in these populations.
- Assess the relationships between socio-demographic data and levels of job engagement, resiliency, and the Five Facet Mindfulness among NICU and PICU nurses.
- Evaluate the differences between the two settings in terms of study variables, including levels of job engagement, resiliency, and five-facet meditations.

#### Hypotheses

- H1: Mindfulness training will significantly enhance emotional resilience, job engagement, and psychological outcomes, as measured by the FFMQ, among NICU and PICU nurses in Saudi Arabia and Egypt.
- H2: No significant differences will be observed in the effectiveness of mindfulness training on emotional resilience, job engagement, and FFMQ outcomes between Saudi and Egyptian nurses.
- H3: Cultural factors and demographic characteristics moderate the impact of mindfulness training on emotional resilience, job engagement, and FFMQ outcomes, potentially contributing to differences between Saudi and Egyptian nurses.

#### Research questions

- What are the baseline levels of emotional resilience, job engagement, and psychological well-being (as assessed by the FFMQ) among NICU and PICU nurses in Saudi Arabia and Egypt?
- What changes in emotional resilience, job engagement, and FFMQ-assessed psychological well-being were observed after mindfulness training?
- How does the effect of mindfulness training on emotional resilience, FFMQ-measured psychological well-being, and job engagement differ between NICU and PICU nurses in Saudi Arabia and Egypt?
- Which cultural factors influence the outcomes of mindfulness training on emotional resilience, FFMQ-assessed psychological well-being, and job engagement in these populations?

## Materials and methods

### Study subjects and settings

Data were collected from two tertiary pediatric hospitals: King Abdullah Specialist Children’s Hospital (KASCH) in Jeddah, Saudi Arabia, and the Pediatric Hospital of Mansoura University in Egypt. Each institution functions as a major center for the referral of both neonatal and pediatric patients requiring critical care. Additionally, both hospitals provide advanced clinical services congruent with those offered by NICU/PICU.

### The king Abdullah specialist children’s hospital, Saudi Arabia

KASCH is a tertiary children’s hospital located in Jeddah, Saudi Arabia. The hospital has an approximate capacity of over 300 beds and provides a comprehensive array of medical specialties within the field of pediatrics, including Neonatology, Pediatric Critical Care, Pediatric Cardiology, Pediatric Oncology, and Emergency Medicine. The KASCH Neonatal Intensive Care Unit (NICU) is an approximately sixty-five-bed facility that routinely provides care for acute, high-risk neonatal patients transferred from the western region of Saudi Arabia. The KASCH Pediatric Intensive Care Unit (PICU) has approximately 25 beds and provides intensive care nursing for pediatric patients with medical and surgical conditions. Most nursing staff at KASCH are bachelor-prepared Nurse Practitioners and work within a standardized, team-based model of care consistent with the standards set forth by the National Guard Health Affairs (NGHA) regarding Quality and Patient Safety Accreditation. Due to the volume of patients seen and the complexity of cases in both units, KASCH has adopted an innovative technology-driven approach to the provision of Critical Care Nursing.

### Mansoura university pediatric hospital, Egypt

With over 200 beds across its departments, the Pediatric Hospital at Mansoura University is both an academic and referral center. This facility offers multiple pediatric subspecialties, including neonatal intensive care (NICU), pediatric intensive care unit (PICU), nephrology, cardiology, and oncology. The NICU has approximately 45 beds and provides care for many premature and critically ill newborns from the Dakahlia Governorate and surrounding areas. The PICU has approximately 20 beds and provides care for a wide variety of medical and surgical conditions affecting pediatric patients. The nursing staff at this facility are full-time employees with diploma and/or bachelor’s qualifications; they work according to nationally recognized standards and protocols for providing critical care.

### Comparability of study settings

The study sites differed in numerous ways, including the size of the facilities, the structure of administrative operations, and the type of accreditations received. Despite these differences, the NICU and PICU departments of the two hospitals are functionally comparable. Each unit is designed to provide tertiary-level critical care for patients with the same level of acuity, using similar staffing patterns and facing similar clinical demands. Thus, the similarities between the two units serve as a strong foundation for comparing the impact of mindfulness training on emotional resilience and job engagement among nurses in the two countries.

### Study design

A quasi-experimental, non-randomized design technique was used in this research project to show the effects of a behavioral intervention for nurses in Egypt and Saudi Arabia. The lack of a randomized controlled trial design means that this study does not fit the definition of a clinical trial and, therefore, does not have an assigned clinical trial number. Before data collection, ethical approval was obtained from the appropriate institutional review boards, and all participants provided informed consent to participate in the study. The growing capacity of healthcare institutions to collect routine clinical data has led to the increased use of quasi-experimental designs in medical informatics and other medical disciplines (39). All eligible participants from Egypt and Saudi Arabia were assigned to the intervention group, and outcomes were compared pre-{{-}}- and post-intervention, within and between the two national cohorts. Because randomization and a control group were not included, confounding variables could not be eliminated. To minimize potential bias, consistent inclusion and exclusion criteria were applied across both sites, and baseline characteristics were assessed to ensure comparability between the groups.

### Sample size calculation

To ensure that the study was adequately powered, 148 nurses were initially approached, 80 from Mansoura University Pediatric Hospital (Egypt) and 68 from King Abdullah Specialized Children’s Hospital (KASCH, Saudi Arabia). After screening for eligibility, 120 nurses (60 from each country) met the inclusion criteria and participated in the study, while 28 nurses [20 from Egypt and 8 from Saudi Arabia] did not meet the inclusion criteria.

In addition to this screening-based estimation, a formal *post-hoc* power analysis was conducted using G*Power software [version 3.1.9.7]. Assuming a medium effect size (Cohen’s d = 0.5), significance level of α = 0.05, and a two-tailed independent t-test with 60 participants per group, the *post-hoc* power was calculated to be approximately 0.87 [87%]. This confirmed that the planned sample size was sufficient to detect medium effect sizes with adequate statistical power.

The initial Rao soft calculation for the sample size was based on the following formula:


x = Z(c/100)2r(100−r)

n = N x/((N−1)E2 + x)

E = Sqrt[(N − n)x/n(N−1)]

**Table 1** outlines the recruitment and eligibility of participants at the two study locations. Out of 148 individuals approached, 120 satisfied the eligibility requirements and were included in the final analysis. At Mansoura University Pediatric Hospital in Egypt, 60 out of 80 approached participants were deemed eligible, resulting in 20 exclusions. Similarly, at King Abdullah Specialist Children’s Hospital in Saudi Arabia, 60 of the 68 participants met the inclusion criteria, with 8 being excluded. A post-hoc power analysis, with an alpha level of 0.05 and a moderate effect size (d = 0.5), revealed a statistical power of 0.87 for both sites, confirming that the sample size was sufficient for the intended analyses. The combination of survey methods and power analysis methodologies guarantees that appropriate sample sizes will be achievable for statistical validity.

**Table 1:** Sample size and *post-hoc* power analysis.

Country/Hospital	Total approached	Eligible & included	Not eligible	*Post-hoc* power (α = 0.05, d = 0.5)
Egypt (Mansoura University Pediatric Hospital)	80	60	20	0.87
Saudi Arabia (KASCH)	68	60	8	0.87
Total	148	120	28	0.87

### Sampling technique

A purposive convenience sampling method was used to recruit NICU and PICU nurses from hospitals (Mansoura Pediatric Hospital and KASCH) with similar demographics and care standards. Nurses who met the inclusion criteria were assigned to the intervention group, ensuring comparability between the pre- and post-training assessments.

#### CONSORT 2025 flow diagram for participant enrollment

**Figure 2:**
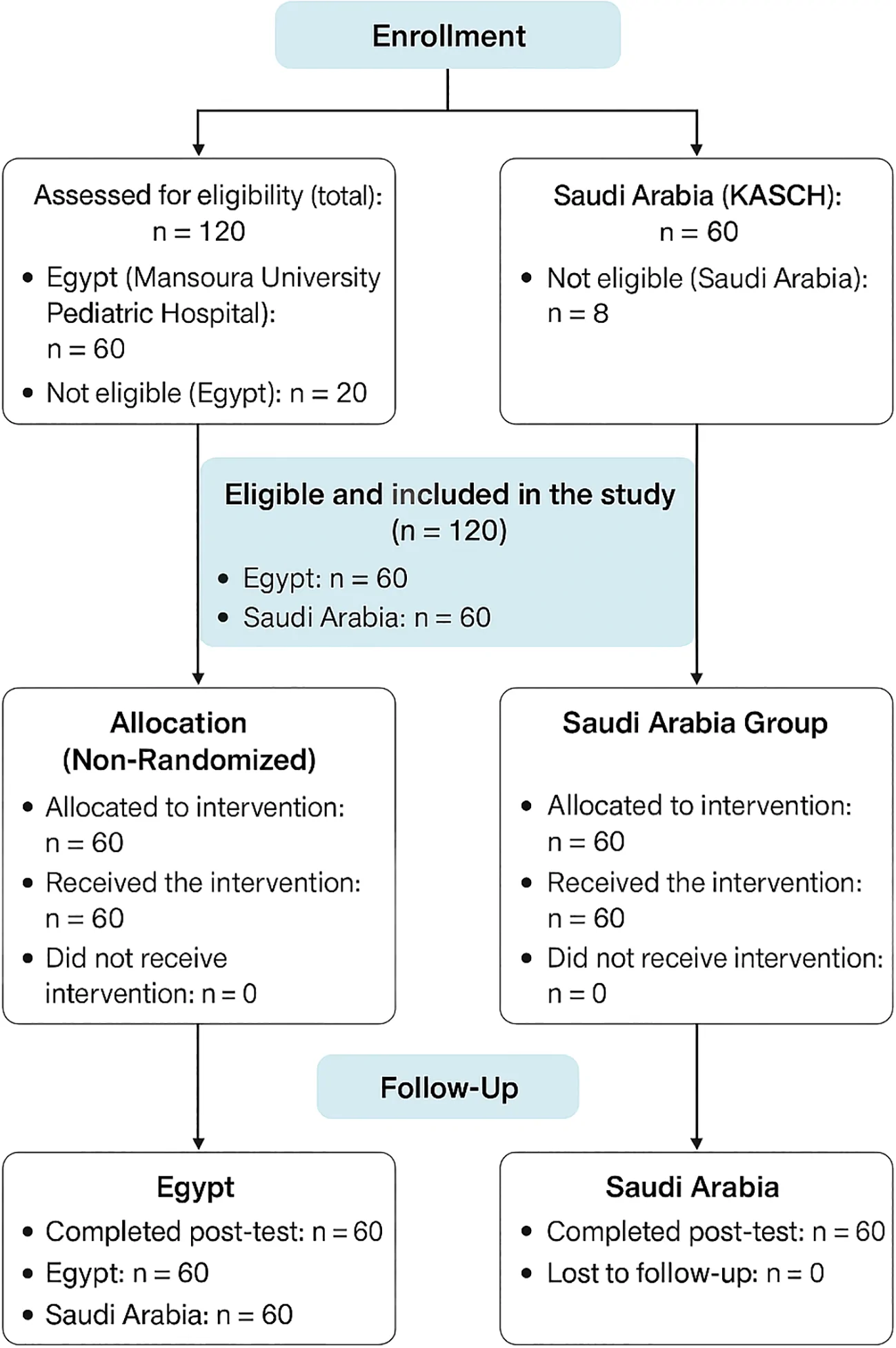
CONSORT 2025 flow diagram (extension for non-randomized studies) showing participant recruitment, eligibility screening, allocation, follow-up, and analysis across NICU and PICU nurses in Egypt and Saudi Arabia.

**Figure 2** outlines a well-structured study involving participants from Egypt and Saudi Arabia, with 120 individuals assessed for eligibility (60 from each country), resulting in 20 exclusions in Egypt and 8 in Saudi Arabia. Despite these exclusions, both sites contributed to 60 eligible participants, suggesting that additional recruitment was required to maintain balance. All participants were non-randomly allocated to receive the intervention, with full adherence and no dropouts noted. Follow-up was robust, with every participant completing the post-test, and no losses were reported, indicating strong engagement and data integrity. However, the absence of randomization and a control group may limit the ability to draw causal conclusions.

### Data collection instruments

To achieve the objective of this study, four main tools were used.

1. Demographic data of the participants, this part will ask about nurses’ age, gender, marital status, educational level, and years of experience in general and specifically in the NICU and PICU departments.

2. Connor-Davidson Resilience Scale (CD-RISC)

The Connor-Davidson Resilience Scale (CD-RISC), developed by Connor and Davidson (40) in 2003, is a widely used self-report assessment tool designed to evaluate adult resilience. It measures an individual’s capacity to effectively manage stress and adversity across multiple dimensions, including personal competence, trust in one’s instincts, tolerance of negative affect, and positive acceptance of change. Typically comprising 25 items, the CD-RISC also offers shorter versions, with respondents rating each item on a 5-point Likert scale. The scores were summed to calculate the total resilience score, ranging from 0 to 100, with higher scores indicating greater resilience. Sample items include statements like “I can adapt when changes occur” and “I tend to bounce back after illness or hardship.” The CD-RISC has demonstrated reliability and validity across diverse populations and cultural contexts, showing high internal consistency and positive correlations with measures of psychological well-being, and negative correlations with measures of distress and psychopathology. It is utilized in clinical practice to assess resilience levels in individuals facing various challenges or stressors and in research settings to explore resilience as a protective factor against mental health difficulties and evaluate the effectiveness of interventions aimed at enhancing resilience.

3. Utrecht Work Engagement Scale (UWES)

The Utrecht Work Engagement Scale (UWES), developed by Schaufeli, Bakker, and Salanova (41) in 2002, is a widely used self-report instrument for measuring work engagement. Work engagement refers to a positive, fulfilling, work-related state of mind, characterized by vigor, dedication, and absorption. This scale assesses the extent to which individuals are engaged in their work and experience a sense of energy, enthusiasm, and absorption in their tasks.

- Vigor: Energy, enthusiasm, and resilience experienced while working.
- Dedication: A strong sense of significance, pride, and enthusiasm toward one’s work.
- Absorption: Being fully immersed in work to the point of losing track of time.

The UWES typically consists of 17 items rated on a 7-point Likert scale (0 = never to 6 = always). The scores for each dimension were calculated by summing the responses to the corresponding items. Higher scores indicate higher levels of work engagement in the study. The UWES has demonstrated strong reliability and validity across occupational and cultural contexts, correlating positively with job satisfaction, organizational commitment, and performance.

4. Five Facet Mindfulness Questionnaire (FFMQ)

The Five Facet Mindfulness Questionnaire (FFMQ), developed by Baer et al. (42), is a comprehensive tool designed to evaluate mindfulness across five key facets: observing, describing, acting with awareness, non-judging of inner experience, and non-reactivity to the inner experience. It was created by integrating items from existing questionnaires on mindfulness. The FFMQ has been extensively validated by Baer et al. (43), Veehof et al. (44), and Gu et al. (45), showing strong construct, criterion, and predictive validity, and high internal consistency (Cronbach’s α = 0.75–0.91).

• Scoring:

- *Observing*: Items 1–7.
- *Describing*: Items 8–13 (11–13 reverse-scored).
- *Acting with Awareness*: Items 14–20 (14, 16–20 reverse-scored).
- *Non-judging of Inner Experience*: Items 21–26 (all reverse-scored).
- *Non-reactivity to Inner Experience*: Items 27–30.

Reverse-scored items should be adjusted before being summed. Higher scores indicate greater levels of mindfulness. Scores can be categorized as high, moderate, or low using normative data or sample distributions.

### Validity and reliability

Translation and back-translation procedures were conducted to ensure the accuracy of the instruments, as the participants’ primary language was Arabic. To establish content validity, the Arabic versions of the scales were reviewed by a panel of five nursing experts who provided feedback and suggestions that were incorporated into the final instruments. A pilot study involving 10% of the target sample was conducted to evaluate the language clarity, ease of understanding, and questionnaire completion time. The Cronbach’s alpha value of the Complete Five Facet Mindfulness Questionnaire is 0.902, and of the Utrecht Work Engagement Scale is 0.896, and of the CD-RISC Resiliency scale is 0.898

### Data collection procedure

Permission to conduct the study was obtained from the CONJ Research Unit, KAIMRC IRB, and the managers of the two hospitals in Egypt and Saudi Arabia. Participants were provided with detailed information about the study’s purpose and provided written informed consent before participation.

The primary objective of the mindfulness training program was to cultivate mindfulness and help participants develop greater awareness, acceptance, and resilience when facing life’s challenges. The program provides practical tools and techniques aimed at reducing stress, enhancing well-being, and promoting personal growth.

### Data collection phases

Data collection was conducted in three main phases.

1. Introduction and informed consent

After study approval, participants completed a demographic questionnaire that included items on age, gender, and professional experience. They also completed pre-assessment tools to measure baseline levels of mindfulness, emotional resilience, and job engagement after receiving an overview of the study and signing an informed consent form.

### Implementation of the program

The participants were divided into smaller groups of 10–15 nurses to ensure more personalized and interactive sessions. A schedule was organized based on nurse availability, with training conducted during working hours from 8:00 a.m. to 4:00 p.m. Various instructional methods were employed, including PowerPoint presentations, brainstorming sessions, demonstrations, videos, brochures, and practical examples. Each session concluded with a summary of the skills learned, and the participants received brochures containing the training materials.

During data collection, several challenges were encountered, primarily due to the busy schedules and high workload of NICU and PICU nurses, which limited their availability to attend all the training sessions. To address this barrier, video recordings of the mindfulness sessions were developed and distributed to participants, allowing them to watch the sessions at their convenience before completing post-test assessments. This approach ensured that all participants had access to the full program content despite scheduling constraints and helped to maintain the integrity of the intervention. In addition, to ensure that both sites would be adhering to a common and equalized delivery of the intervention, there was a standardized protocol was developed and implemented consistently across both sites. All facilitators were given the same training, and all facilitators had access to the same training materials. The actual intervention sessions (face-to-face and video) at both sites contained the same content, timing, and order; fidelity checks were the same, and supervision of facilitators was conducted periodically to ensure that participants from both sites had access to all core components of the intervention. These procedures were put in place to reduce differences and increase the ability to compare results across both sites.

**Figure 3:**
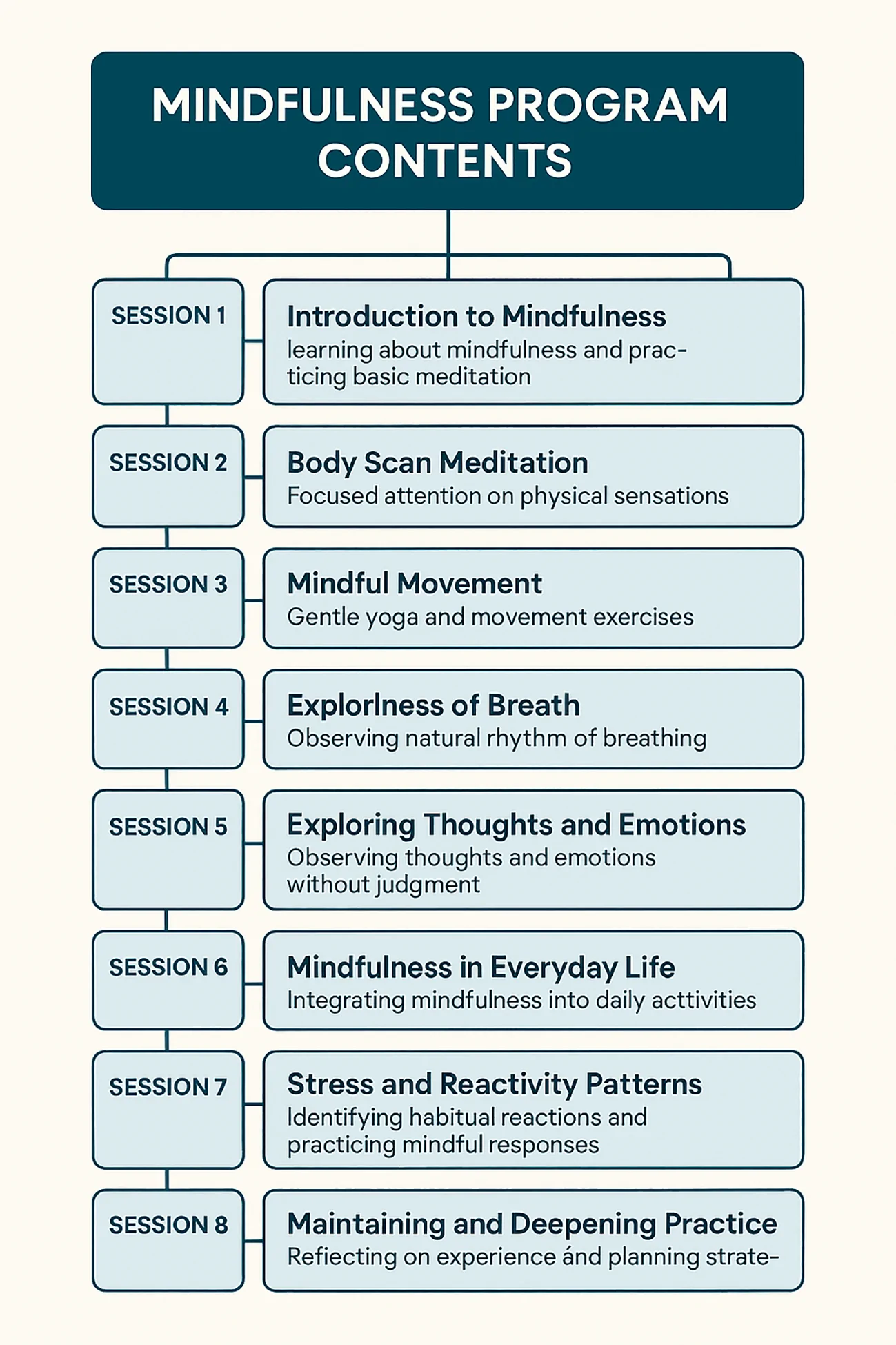
Mindfulness sessions’ contents, as they were delivered to the participants.

The Mindfulness-Based Stress Reduction (MBSR) program, originally developed by Dr. Jon Kabat-Zinn (46) in 1979, was adapted for NICU and PICU nurses in this study. The program consisted of eight weekly sessions of approximately 2.5–3 hours each, with a full-day retreat between sessions six and seven. The key components included in **Figure 3** *are as follows:*

Participants engaged in formal practices (sitting meditation, body scan, and mindful movement) and informal practices to incorporate mindfulness into their daily lives. Group discussions and experiential exercises facilitated reflections, sharing, and peer learning.

2. Closing and post-assessment

Upon completion of the program, the participants completed post-assessment tools to evaluate the effectiveness of the intervention.

The mindfulness training program was delivered by the PI (prof Amal I. Khalil), who is certified to practice mindfulness as well as receive training from Alison Training Center, USA. By being trained by an organization specifically focused on teaching the techniques of mindfulness, the PI met the qualification requirements necessary to implement the mindfulness intervention. Conducting the mindfulness intervention by the same qualified individual assured the continuity and fidelity of this intervention across all participants.

### Data management and analysis

The assumptions of normality (Shapiro-Wilk), homogeneity of variances (Levene’s), and absence of multicollinearity (Variance Inflation Factor) were checked prior to conducting all statistical analyses. Effect sizes (Cohen’s d) were recalculated employing corrected pooled standard deviations for accuracy. Additionally, to descriptive statistics and bivariate comparisons, to remove possible demographic confounding effects due to age, sex, years of experience, level of qualification, and type of unit, a multiple linear regression model was used to control these possible confounders and assess the independent effect of the intervention on the post-test scores controlling predefined baseline characteristics.

The adequacy of the regression model was assessed using residual plots, VIFs, tolerance values, and Cook’s distance. The statistical analyses were performed using SPSS version 20 and a significance level of p < 0.05 was established.”

## Results

**Table 2** shows a comparison of social and personal backgrounds for Egyptian and Saudi nurses. Nurses’ profiles across both countries were very similar. There were no significant differences between the two groups in any aspect. Most nurses were between the ages of 25-44. Saudi Arabia had slightly more nurses aged 45-54. Nurses were mostly female in both Egypt and Saudi Arabia. Saudi Arabia had somewhat more female nurses. Nurses in both countries had very similar education levels. Saudi nurses were more likely to hold diplomas. Egyptian nurses had more master’s degrees. Nursing experience and years spent in the NICU/PICU were also similar. Saudi nurses appeared to have somewhat higher nursing experience. Most nurses worked as staff nurses. Head nurses were the second most common. Nurse practitioners were the least common. Nurses in both countries generally worked full-time. The most common schedule was rotating shifts, with Saudi nurses representing the majority. Most Saudi nurses were single. More Egyptian nurses were married. Saudi nurses had fewer children, which aligns with marital status trends. Very few nurses reported having prior mindful training in either country. Job satisfaction was generally at a neutral or positive level. Very few nurses reported being unhappy. Egyptian nurses were slightly more likely to be unhappy, but still reported some level of job satisfaction. Egyptian nurses were slightly more likely to volunteer for their roles. Saudi nurses were more likely to be assigned by management. These differences were not significant. The overall lack of major differences suggests the two groups are comparable. This allows for a meaningful study comparing the nurses from both nations.

**Table 2:** Socio-demographic characteristics of the study sample by study location (Egypt and Saudi Arabia) (n = 120).

	Egypt		Saudi		Chi – square/fisher’s exact test	
	n	%	n	%	X^2^	P
Age (Years)						
< 25	3	5.0	5	8.3		
25 – 34	30	50.0	25	41.7		
35 – 44	20	33.3	17	28.3		
45 – 54	6	10.0	11	18.3		
> 54	1	1.7	2	3.3	3.002	0.558
Gender						
Male	14	23.3	8	13.3		
Female	46	76.7	52	86.7	2.004	0.157
Educational level						
Diploma in Nursing	2	3.3	8	13.3		
Bachelor’s degree in nursing	52	86.7	48	80.0		
Master’s degree in nursing	6	10.0	4	6.7	4.160	0.125
Years of Experience in Nursing						
1 – 3	5	8.3	9	15.0		
4 – 6	21	35.0	14	23.3		
7 – 10	15	25.0	12	20.0		
> 10	19	31.7	25	41.7	3.694	0.296
Years of Experience in NICU/PICU						
1 – 3	5	8.3	12	20.0		
4 – 6	21	35.0	12	20.0		
7 – 10	18	30.0	18	30.0		
> 10	16	26.7	18	30.0	5.455	0.141
Occupation						
Staff Nurse	30	50.0	34	56.7		
Head Nurse	24	40.0	18	30.0		
Nurse Practitioner	6	10.0	8	13.3	1.393	0.498
Employment Status						
Full-time	41	68.3	47	78.3		
Part-time	19	31.7	13	21.7	1.534	0.215
Work Schedule						
Day shift	8	13.3	3	5.0		
Night shift	14	23.3	11	18.3		
Rotating shifts	38	63.3	46	76.7	3.395	0.183
Marital Status						
Single	20	33.3	31	51.7		
Married	36	60.0	27	45.0		
Divorced	3	5.0	2	3.3		
Widowed	1	1.7	0	0.0	4.858	0.182
Number of Children						
None	27	45.0	39	65.0		
1	7	11.7	5	8.3		
2	14	23.3	9	15.0		
3	8	13.3	5	8.3		
4 or more	4	6.7	2	3.3	4.961	0.291
Previous Mindfulness						
No	45	75.0	47	78.3		
Yes	15	25.0	13	21.7	0.186	0.666
Level of Job Satisfaction						
Very dissatisfied	0	0.0	2	3.3		
Dissatisfied	7	11.7	4	6.7		
Neutral	26	43.3	28	46.7		
Satisfied	24	40.0	22	36.7		
Very satisfied	3	5.0	4	6.7	3.122	0.538
Way of Employment						
Volunteered for the position	34	56.7	29	48.3		
Assigned by management	26	43.3	31	51.7	0.835	0.361

**Figure 4:**
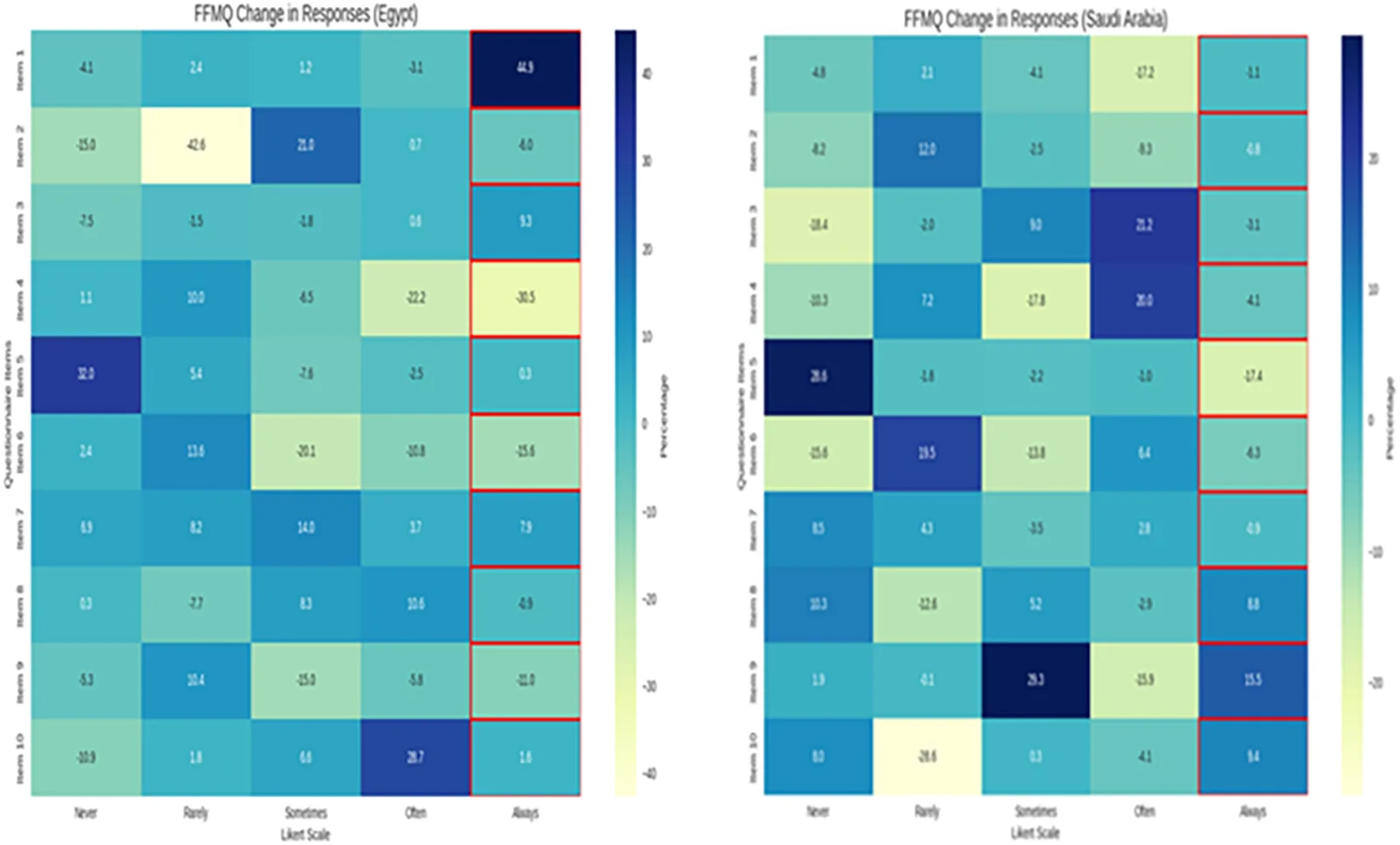
Visual summary of the five facet mindfulness questionnaire results across Egypt and Saudi Arabia pre- and post-intervention.

**Figure 4** present the Five Facet Mindfulness Questionnaire findings, which indicated that the Egyptian participants had larger improvements in nearly every dimension of mindfulness when compared to the Saudi Arabian participants. The Egyptian participants showed notable improvements in expressing emotion, sensory awareness, and non-reactivity to distressing thoughts. For example, items such as “I’m good at finding words that describe my feelings” and “I simply notice distressing thoughts, and they leave,” demonstrated a prevalent shift to higher levels of mindfulness and demonstrated strong statistical significance. While it was promising to have changes in a more positive direction in the Saudi Arabian participants, particularly with sensory engagement and some aspects of emotional regulation, they appeared to have a more moderate and selective level of improvement from the baseline. The heatmaps supported these trends, where the Egyptian responses were more consistently moving from low to high mindfulness ratings across the five dimensions of mindfulness. Saudi Arabia, on the other hand, had a more conditional and varied pattern. These findings suggest that the mindfulness intervention had a greater effect on the participants in Egypt. This could be due to cultural influence, intervention delivery, or participants having different levels of awareness regarding their own mindfulness, thoughts, and feelings.

According to **Table 3**, Egyptian nurses achieved a significant increase in their levels of mindfulness after participating in this study. The overall score on the FFMQ scale for Egyptian nurses rose from a mean value of 74.8 ± 19.6 before the intervention to 164.0 ± 10.5 after the intervention. In comparison, Saudi nurses reported an increase in their FFMQ scores from a mean value of 79.7 ± 20.5 before the intervention to 136.5 ± 18.1 after the intervention. The effect sizes were exceptionally large for both groups: Egyptian nurses’ = 5.33 and Saudi nurses’ = 2.91. Furthermore, 91.7% of Egyptian nurses reached a high level of mindfulness, whereas only 25.0% of Saudi nurses did so. In addition, **Table 4** shows the change in work engagement (as measured by the UWES scale): Egyptian nurses’ total UWES score increased from a mean value of 24.2 ± 10.3 pre-intervention to 81.3 ± 9.5 post-intervention, while Saudi nurses’ total score increased from a mean value of 26.1 ± 10.2 pre-intervention to 72.6 ± 15.3 post-intervention. High levels of work engagement were reached by 91.7% of Egyptian nurses and 73.3% of Saudi nurses, making the effect size for Egyptian nurses (d = 6.34) larger than that for Saudi nurses (d = 3.77). **Table 5** indicates improvements in resilience; specifically, Egyptian nurses’ resilience scores increased from a mean of 13.1 ± 6.7 pre-intervention to 36.3 ± 6.5 post-intervention, whereas Saudi nurses’ scores increased from a mean of 14.7 ± 6.3 pre-intervention to 28.7 ± 6.4 post-intervention. High levels of resilience were found among 85.0% of Egyptian nurses compared with 66.7% of Saudi nurses, with effect sizes of 3.51 and 2.21, respectively. Overall, these data suggest that both Egyptian and Saudi nurses benefited significantly from the intervention, but Egyptian nurses experienced greater improvements than Saudi nurses across all outcomes (mindfulness, work engagement, and resilience).

**Table 3:** Comparison of mindfulness, work engagement, and resilience scores among egyptian and saudi nurses (n = 120).

Measure & domain	Egypt (Mean ± SD/n, %)	Saudi (Mean ± SD/n, %)	Test statistic	p-value	Effect size (cohen’s d)
FFMQ – Pre-Intervention					
Observing	13.7 ± 4.3	14.8 ± 4.3	t = 1.401	0.164	—
Describing	11.2 ± 3.7	11.9 ± 4.0	t = 0.995	0.322	—
Acting with Awareness	13.7 ± 4.0	14.7 ± 4.1	t = 1.352	0.179	—
Non-judging	14.8 ± 4.6	15.9 ± 4.5	t = 1.324	0.188	—
Non-reactivity	21.4 ± 5.8	22.4 ± 6.5	t = 0.889	0.376	—
Total Score	74.8 ± 19.6	79.7 ± 20.5	t = 1.338	0.183	—
FFMQ – post-intervention					
Observing	29.8 ± 3.0	27.2 ± 6.0	t = 3.002	0.003*	Egypt: 4.00, Saudi: 2.06
Describing	25.7 ± 2.4	21.4 ± 3.5	t = 7.849	<0.001**	Egypt: 4.65, Saudi: 2.52
Acting with Awareness	30.4 ± 2.7	26.6 ± 5.2	t = 5.024	<0.001**	Egypt: 4.41, Saudi: 2.88
Non-judging	30.2 ± 2.7	24.6 ± 5.8	t = 6.780	<0.001**	Egypt: 3.36, Saudi: 1.96
Non-reactivity	47.8 ± 4.1	36.7 ± 7.0	t = 10.599	<0.001**	Egypt: 5.02, Saudi: 2.42
Total Score	164.0 ± 10.5	136.5 ± 18.1	t = 10.180	<0.001**	Egypt: 5.33, Saudi: 2.91
**FFMQ Mindfulness Level – Post**			χ² = 54.857	<0.001**	—
Low	0 (0.0%)	0 (0.0%)			
Moderate	5 (8.3%)	45 (75.0%)			
High	55 (91.7%)	15 (25.0%)			
UWES – Pre-Intervention					
Vigor	8.0 ± 3.2	8.5 ± 2.4	t = 0.968	0.335	—
Dedication	8.3 ± 2.4	9.2 ± 3.9	t = 1.522	0.131	—
Absorption	8.0 ± 2.9	8.5 ± 3.0	t = 0.928	0.355	—
Total Score	24.2 ± 10.3	26.1 ± 10.2	t = 1.015	0.312	—
UWES – post-intervention					
Vigor	28.4 ± 4.0	24.6 ± 6.0	t = 4.082	<0.001**	Egypt: 5.71, Saudi: 3.52
Dedication	24.3 ± 2.9	24.1 ± 4.9	t = 0.272	0.786	Egypt: 7.42, Saudi Arabia: 3.82
Absorption	28.6 ± 4.1	24.0 ± 6.9	t = 4.439	<0.001**	Egypt: 5.94, Saudi: 3.73
Total Score	81.3 ± 9.5	72.6 ± 15.3	t = 3.742	<0.001**	Egypt: 6.34, Saudi: 3.77
**UWES Engagement Level – Post**			χ² = 8.105	0.017*	—
Low	0 (0.0%)	4 (6.7%)			
Moderate	5 (8.3%)	12 (20.0%)			
High	55 (91.7%)	44 (73.3%)			
**Resilience – Pre-Intervention**			χ² = 0.152	0.697	—
Low	57 (95.0%)	56 (93.3%)			
High	3 (5.0%)	4 (6.7%)			
Mean ± SD	13.1 ± 6.7	14.7 ± 6.3	t = 1.348	0.180	—
**Resilience – post-intervention**			χ² = 5.502	0.019*	—
Low	9 (15.0%)	20 (33.3%)			
High	51 (85.0%)	40 (66.7%)			
Mean ± SD	36.3 ± 6.5	28.7 ± 6.4	t = 6.454	<0.001**	Egypt: 3.51, Saudi: 2.21

*Statistically significant at p < 0.05 **Statistically significant at p < 0.01.

Effect Size Interpretation: Small effect: d ≈ 0.2; Moderate effect: d ≈ 0.5; Large effect: d ≥ 0.8; Very large effect: d ≥ 1.5 (used in behavioral sciences).

**Table 4:** Association between sociodemographic characteristics and mindfulness levels (pre- and post-intervention) in Egypt and Saudi Arabia (n = 120).

Variable	Egypt Pre	Egypt Post	Chi-square/p-value (Egypt)	Saudi Pre	Saudi Post	Chi-square/p-value (Saudi)
1. Age (Years)	Low: 53 Mod: 7 High: 0	Mod: 5 High: 55	8.895/0.064	Low: 53 Mod: 6 High: 1	Mod: 45 High: 15	15.707/<0.001**
2. Gender	Male: 12 Female: 41	Male: 2 Female: 43	0.122/0.727	Male: 7 Female: 46	Male: 7 Female: 45	0.769/0.380
3. Educational Level	Dip: 2 Bach: 45 Master: 6	Dip: 0 Bach: 5 Master: 0	1.219/0.544	Dip: 7 Bach: 43 Master: 3	Dip: 8 Bach: 48 Master: 4	1.444/0.486
4. Years of Experience in Nursing	1–3: 4 4–6: 19 7–10: 14 >10 16	1–3: 3 4–6: 2 7–10: 0 >10: 0	1.108/0.775	1–3: 9 4–6: 11 7–10: 10 >10 23	1–3: 0 4–6: 0 7–10: 1 >10 14	22.258/<0.001**
5. Years of Experience in NICU/PICU	1–3: 4 4–6: 19 7–10: 17 >10 13	1–3: 3 4–6: 2 7–10: 0 >10: 0	1.862/0.602	1–3: 12 4–6: 10 7–10: 15 >10 16	1–3: 0 4–6: 0 7–10: 2 >10 13	31.259/<0.001**
6. Occupation	Staff: 26 Head: 21 NP: 6	Staff: 5 Head: 0 NP: 0	0.889/0.641	Staff: 29 Head: 17 NP: 7	Staff: 33 Head: 10 NP: 2	23.120/<0.001**
7. Employment Status	Full: 37 Part: 16	Full: 5 Part: 0	0.459/0.498	Full: 43 Part: 10	Full: 34 Part: 11	3.359/0.186
8. Work Schedule	Day: 7 Night: 12 Rotating: 34	Day: 0 Night: 0 Rotating: 5	0.147/0.929	Day: 2 Night: 9 Rotating: 42	Day: 1 Night: 10 Rotating: 34	4.292/0.117
9. Marital Status	Single: 18 Married: 32 Divorced: 2 Widowed: 1	Single: 2 Married: 3 Divorced: 0 Widowed: 0	1.563/0.668	Single: 27 Married: 24 Divorced: 2 Widowed: 0	Single: 27 Married: 16 Divorced: 2 Widowed: 0	6.654/0.036*
10. Number of Children	None: 24 1: 6 2: 13 3: 6 4+: 4	None: 3 1: 0 2: 2 3: 0 4+: 0	2.241/0.692	None: 34 1: 5 2: 8 3: 5 4+: 1	None: 31 1: 4 2: 6 3: 3 4+: 1	5.245/0.731
11. Previous Mindfulness Training	No: 39 Yes: 14	No: 5 Yes: 0	0.485/0.486	No: 41 Yes: 12	No: 39 Yes: 6	7.365/0.007*
12. Job Satisfaction	Very Diss: 0 Diss: 7 Neutral: 24 Satisfied: 20 Very Sat: 2	Very Diss: 0 Diss: 0 Neutral: 2 Satisfied: 3 Very Sat: 0	3.272/0.352	Very Diss: 1 Diss: 4 Neutral: 25 Satisfied: 20 Very Sat: 3	Very Diss: 2 Diss: 4 Neutral: 22 Satisfied: 14 Very Sat: 3	18.527/0.018*
13. Way of Employment	Voluntary: 29 Assigned: 24	Voluntary: 4 Assigned: 1	0.703/0.402	Voluntary: 27 Assigned: 26	Voluntary: 24 Assigned: 21	1.802/0.179

*Statistically significant at p < 0.05 **Statistically significant at p < 0.01

**Table 5:** Association between sociodemographic characteristics and work engagement levels (pre- and post-intervention) in Egypt and Saudi Arabia (n = 120).

Variable	Egypt Pre	Egypt Post	Chi-square/p-value (Egypt)	Saudi Pre	Saudi Post	Chi-square/p-value (Saudi)
1. Age (Years)	Low: 56 Mod: 4	Mod: 5 High: 55	1.339/0.855	Low: 56 Mod: 3 High: 1	Low: 4 Mod: 15 High: 41	10.644/0.223
2. Gender	Male: 14 Female: 46	Mod: 2 High: 43	1.304/0.253	Male: 8 Female: 51	Low: 1 Mod: 12 High: 37	2.570/0.277
3. Educational Level	Dip: 2 Bach: 48 Master: 6	Dip: 0 Bach: 5 Master: 0	0.659/0.719	Dip: 8 Bach: 44 Master: 4	Low: 0 Mod: 12 High: 42	4.053/0.399
4. Years of Experience in Nursing	1–3: 5 4–6: 21 7–10: 15 >10 19	Mod: 5 High: 55	1.611/0.657	1–3: 8 4–6: 14 7–10: 12 >10 25	Low: 4 Mod: 12 High: 44	23.998/<0.001**
5. Years in NICU/PICU	1–3: 5 4–6: 21 7–10: 18 >10 16	Mod: 5 High: 55	1.055/0.788	1–3: 11 4–6: 12 7–10: 18 >10: 18	Low: 4 Mod: 12 High: 44	32.475/<0.001**
6. Occupation	Staff: 30 Head: 24 NP: 6	Mod: 5 High: 55	0.536/0.765	Staff: 33 Head: 18 NP: 9	Low: 4 Mod: 12 High: 44	16.684/0.002*
7. Employment Status	Full: 41 Part: 19	Mod: 5 High: 55	0.666/0.415	Full: 46 Part: 14	Low: 4 Mod: 12 High: 44	2.285/0.319
8. Work Schedule	Day: 8 Night: 14 Rotating: 38	Mod: 5 High: 55	0.563/0.755	Day: 3 Night: 11 Rotating: 45	Low: 4 Mod: 12 High: 44	4.522/0.340
9. Marital Status	Single: 20 Married: 36 Divorced: 3 Widowed: 1	Mod: 5 High: 55	20.357/<0.001**	Single: 30 Married: 27 Divorced: 2 Widowed: 0	Low: 4 Mod: 12 High: 44	6.868/0.143
10. Number of Children	None: 27 1: 7 2: 14 3: 8 4+: 4	Mod: 5 High: 55	2.219/0.695	None: 38 1: 5 2: 9 3: 5 4+: 2	Low: 4 Mod: 12 High: 44	8.555/0.381
11. Previous Mindfulness	No: 45 Yes: 15	Mod: 5 High: 55	<0.001/1.000	No: 46 Yes: 14	Low: 4 Mod: 12 High: 44	6.035/0.049*
12. Job Satisfaction	Very Diss: 0 Diss: 7 Neutral: 26 Satisfied: 24 Very Sat: 3	Mod: 5 High: 55	7.577/0.056	Very Diss: 2 Diss: 4 Neutral: 28 Satisfied: 21 Very Sat: 4	Low: 4 Mod: 12 High: 44	4.829/0.776
13. Way of Employment	Voluntary: 34 Assigned: 26	Mod: 5 High: 55	0.078/0.781	Voluntary: 29 Assigned: 31	Low: 4 Mod: 12 High: 44	4.757/0.093

*Statistically significant at p < 0.05.

**Statistically significant at p < 0.01.

**Figure 5:**
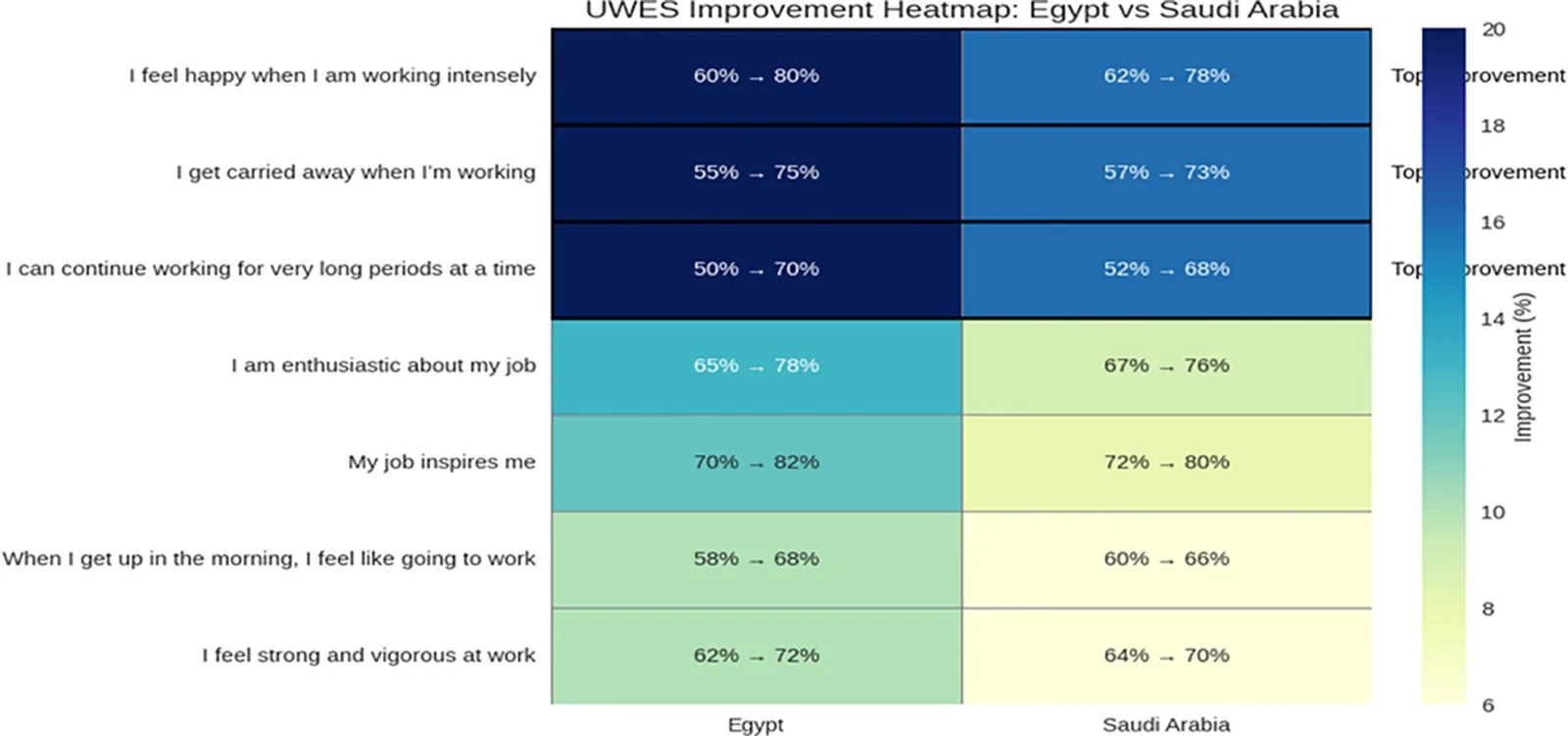
Visual summary of the UWES questionnaire results across Saudi Arabia, pre- and post-intervention

**Figure 5** (Heat Map 3) shows cross-country patterns indicating greater overall improvements in Egypt compared with Saudi Arabia. Nonetheless, both countries exhibited the same top three improved items, suggesting that the mindfulness intervention similarly enhanced emotional engagement and resilience among participants. Items relating to flow and being immersed (e.g., “I get carried away when I’m working”) saw wild shifts from our low preintervention scores to high post-intervention scores. Resilience and stamina (“I can work long periods”) improved significantly after intervention, suggesting that levels of better coping with the demands of the workload were more effective. All-in-all, emotional uplift (“I feel happy when I am working hard”) was the most improved item and suggests greater satisfaction and motivation levels.

**Figure 6:**
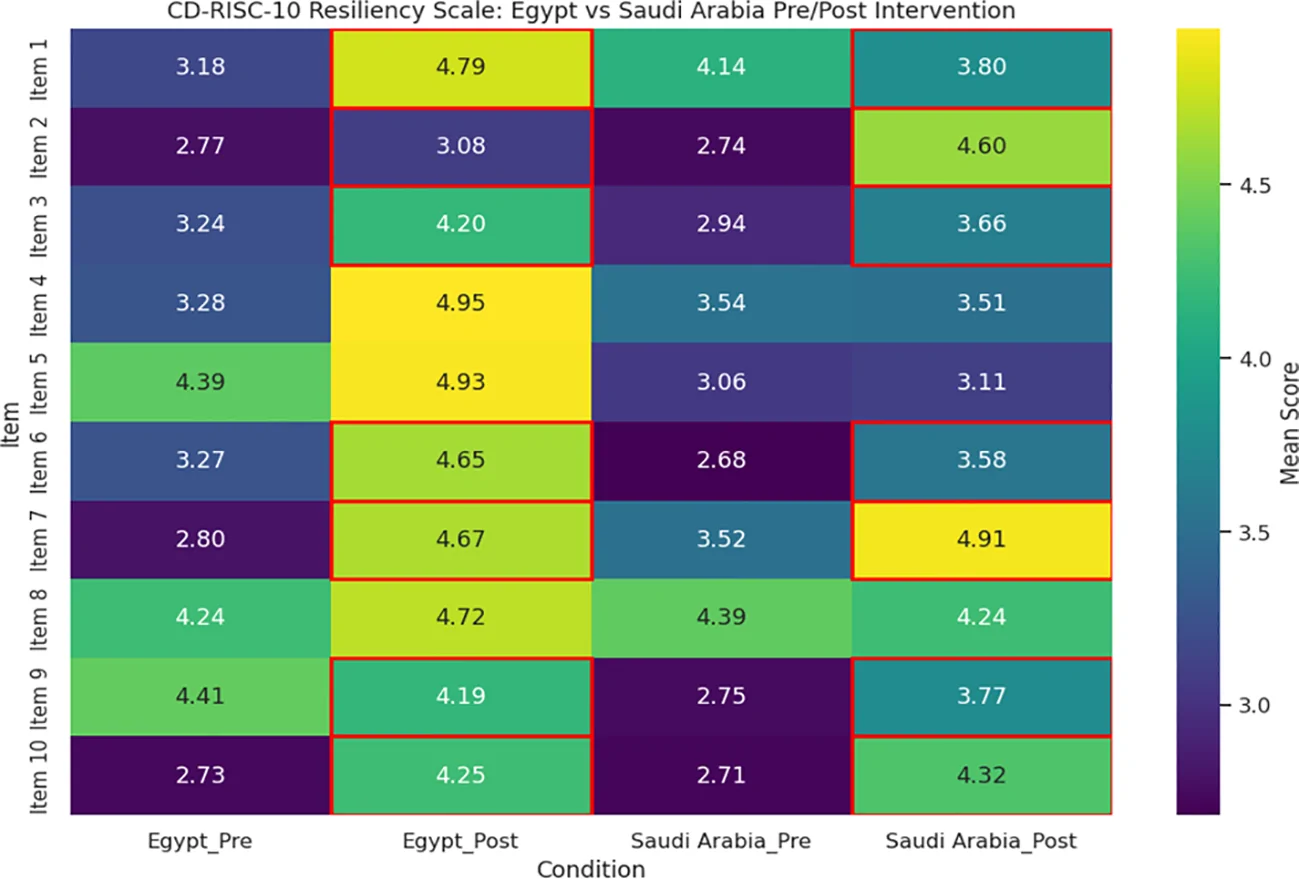
Visual summary of the CD-RISC-10 resiliency scale results across Egypt and Saudi Arabia, pre- and post-intervention. The heatmap highlights mean scores for each item, with red borders marking items that showed statistically significant improvements (p < 0.001) after the intervention.

**Figure 6** proves to be an exceptional visual summary of the results of the CD-RISC-10 Resiliency Scale, showing a clear visual change in resiliency levels after the intervention and once again clearly highlights the change occurring in the Egyptian participants. Before the intervention, responses from both groups were clustered at the lower end of the scale - “rarely true” and “sometimes true” - with the understanding that we were working with a very small sample showing very low baseline resiliency. While the Egyptian nurses moved predominantly to the highest category (“true nearly all the time”) following the intervention, the Saudi participants made a more moderate movement, often ending in “often true.” The cells framed in red reveal statistically significant improvements in each item (p < 0.001), supporting how effective the intervention was. The post-intervention numbers for Egyptian participants in adaptability, emotional regulation, and recovery from adversity showed a strong sense of internalization of the practices presented in the intervention to build resiliency. Again, this visual also substantiates the findings outlined in the quantitative portion of the study and creates a prompt for the reader to think about the context and cultural contours into consideration when discussing possible explanations for why one group improved more than the other. It is an excellent visual to illustrate the effectiveness of the intervention for academic and clinical audiences.

**Table 4** shows the relationship between sociodemographic features and levels of mindfulness for participants in Egypt and Saudi Arabia (pre-intervention), allowing us to visualize the differences between sociodemographic groups at baseline. In Egypt, many of the participants had low levels of mindfulness regardless of their age, gender, education, experience, or employment, as no meaningful statistics could be established, indicating that there was likely a consistent low level of mindfulness across the demographic groups. There were many more distinctions between Saudi nurses, where gender (gender) was significantly related to levels of mindfulness (p = 0.024), in that male nurses tended to report significantly higher levels of mindfulness. Although none of the other characteristics, such as age, experience, and job satisfaction, were found to have meaningful relationships to levels of mindfulness, as indicated by the descriptive distribution, there might still be unknown underlying groups that influence mindfulness and affect potential for change both in general and in response to the mindfulness interventions through our study. Data collected post-intervention indicated meaningful changes in both nations. As indicated, in the case of Egypt, 91.7% of nurses achieved high levels of mindfulness, with no meaningfully related sociodemographic factors identified concluded that intervention was largely effective, and the increase in utilization and engagement was consistent across the sample of nurses in Egypt. In Saudi Arabia, there was more variability reported in the levels of mindfulness, with only 25.0% of nurses achieving high levels, and 75.0% of nurses remaining at moderate levels. Based on sociodemographic factors, there were many meaningful relationships established for nurses in Saudi Arabia post-intervention. Most notably, degree of age (p < 0.001), nursing experience and NICU/PICU experience (p <.001), mindfulness experience (p = 0.007), and job satisfaction (p = 0.018). These findings indicate that although the intervention worked well in both locations, its effectiveness in Saudi Arabia was shaped by personal and professional elements, highlighting the necessity to customize psychological interventions to demographic and experiential backgrounds to enhance results in varied healthcare population**s.**

**Table 5** visually displays the relationship between sociodemographic characteristics and nurse work engagement levels in Egyptian and Saudi nurses before and after the intervention, showing shifts in the dynamics of engagement. Pre-intervention research showed that many nurses in each country reported low engagement (Egypt and Saudi Arabia: 93.3%), and very few variables showed statistically significant relationships. In Saudi Arabia, engagement was significantly related to age (p < 0.001) and marital status (p < 0.001), suggesting that older and widowed nurses may have different initial points of experience relating to work engagement. Post-intervention, both countries saw significant improvements, with Egypt experiencing a marked jump in high engagement (91.7%), and Saudi Arabia experiencing a multiplicative increase (high: 73.3%, moderate: 20.0%). Engagement levels post-intervention indicated a significant relationship with a few factors in both countries. In Egypt, extensive nursing and NICU/PICU experience, significantly associated with increased engagement (p < 0.001), while in Saudi Arabia, significant correlations were found for profession (p = 0.002), prior exposure to mindfulness (p = 0.049), and years of experience (both general and critical care) (p < 0.001). These results indicate that although the intervention was generally successful, its effectiveness was influenced by professional development, type of role, and previous experience with mindfulness, particularly in more hierarchical environments such as Saudi Arabia. The findings highlight the necessity of customizing engagement approaches based on personal and organizational elements to enhance their efficiency among various nursing groups.

**Table 6** summarizes the relationship between sociodemographic factors and resilience among nurses in Egypt and Saudi Arabia at both pre- and post-intervention time points, indicating both the group-level relationship and differences that existed and changed. Before the intervention, a large majority of the nurses in both regions indicated low resilience (Egypt = 95.0%, Saudi Arabia = 93.3%), and they had few sociodemographic associations. In Egypt, and before the intervention, there were no statistically significant associations for the sociodemographic variables, suggesting equally low resilience across age, sex, educational level, and experience baseline. In Saudi Arabia, the significance associated with age (p = 0.016) suggested that older (>54 years) nurses were more likely to have higher resilience. In Egypt, variation for the post-intervention tests indicated significant improvement for both sub-groups, but there were very different patterns of results. In Egypt the findings suggested that 85.0% of nurses achieved high resilience with statistically significant associations for years of nursing experience and NICU/PICU experience (p <.001), occupation (p = .005) where nurses from the same occupation had higher resilience than other occupations, full-time employed (p = .027), and gender (p = .013) suggesting that professional maturity and employed on a full-time basis were related to resilience. In Saudi Arabia the findings indicated that 66.7% of nurses achieved high resilience post-intervention where significant associations were found for age (p = .012), years of experience (p <.001), NICU/PICU tenure (p <.001), occupation (p <.001), mindfulness beforehand (p = .027), work schedule (p = .037). These results indicate that while the intervention was generally effective in both contexts, its impact was influenced by individual and professional factors, especially in Saudi Arabia, where demographic stratification remained more evident. The findings emphasize the need to customize resilience-building strategies to align with nurses’ experiential and occupational backgrounds to enhance psychological outcomes in diverse health care settings.

**Table 6:** Association between sociodemographic characteristics and resilience levels (pre- and post-intervention) in Egypt and Saudi Arabia (n = 120).

Variable	Egypt Pre	Egypt Post	Chi-square/p-value (Egypt)	Saudi Pre	Saudi Post	Chi-square/p-value (Saudi)
1. Age (Years)	Low: 57 High: 3	Low: 9 High: 51	0.702/0.951	Low: 56 High: 4	Low: 20 High: 40	12.798/0.012*
2. Gender	Male: 12 Female: 45	Low: 5 High: 4	3.315/0.069	Male: 8 Female: 48	Low: 20 High: 40	1.803/0.179
3. Educational Level	Dip: 2 Bach: 49 Master: 6	Dip: 0 Bach: 9 Master: 0	1.629/0.443	Dip: 7 Bach: 45 Master: 4	Dip: 1 Bach: 48 Master: 1	2.063/0.357
4. Years of Experience in Nursing	1–3: 5 4–6: 21 7–10: 15 >10 19	Low: 9 High: 51	34.603/<0.001**	1–3: 9 4–6: 14 7–10: 12 >10 25	Low: 20 High: 40	20.131/<0.001**
5. Years in NICU/PICU	1–3: 5 4–6: 21 7–10: 18 >10 16	Low: 9 High: 51	34.603/<0.001**	1–3: 11 4–6: 11 7–10: 17 >10: 17	Low: 20 High: 40	32.500/<0.001**
6. Occupation	Staff: 30 Head: 24 NP: 6	Low: 9 High: 51	10.588/0.005*	Staff: 32 Head: 16 NP: 8	Low: 20 High: 40	18.029/<0.001**
7. Employment Status	Full: 41 Part: 19	Low: 9 High: 51	4.907/0.027*	Full: 44 Part: 12	Low: 20 High: 40	3.142/0.076
8. Work Schedule	Day: 8 Night: 14 Rotating: 38	Low: 9 High: 51	3.181/0.204	Day: 3 Night: 10 Rotating: 47	Low: 20 High: 40	6.578/0.037*
9. Marital Status	Single: 20 Married: 33 Divorced: 3 Widowed: 1	Low: 9 High: 51	1.133/0.769	Single: 29 Married: 25 Divorced: 2 Widowed: 0	Low: 20 High: 40	4.868/0.088
10. Number of Children	None: 27 1: 7 2: 14 3: 8 4+: 4	Low: 9 High: 51	6.384/0.172	None: 38 1: 4 2: 7 3: 5 4+: 2	Low: 20 High: 40	4.538/0.338
11. Previous Mindfulness	No: 45 Yes: 15	Low: 9 High: 51	3.529/0.060	No: 43 Yes: 13	Low: 20 High: 40	4.910/0.027*
12. Job Satisfaction	Very Diss: 0 Diss: 7 Neutral: 26 Satisfied: 24 Very Sat: 3	Low: 9 High: 51	2.081/0.556	Very Diss: 2 Diss: 3 Neutral: 27 Satisfied: 20 Very Sat: 4	Low: 20 High: 40	3.881/0.422
13. Way of Employment	Voluntary: 34 Assigned: 26	Low: 9 High: 51	2.348/0.125	Voluntary: 27 Assigned: 29	Low: 20 High: 40	3.337/0.068

*Statistically significant at p < 0.05

**Statistically significant at p < 0.01.

**Table 7** shows the relative relationships among mindfulness, work engagement, and resilience before and after the intervention, showing differences in the correlation pattern. Before the intervention, in both the Egyptian and Saudi groups, all correlations among the three variables were weak and not statistically significant, suggesting that these constructs were operating separately at the pre-intervention baseline phase. However, when we evaluated the data post-intervention, there was a robust and statistically significant relationship across all three variables. For example, in the Egyptian cohort, mindfulness had a strong relationship to work engagement (r = 0.509, p < 0.001) and resilience (r = 0.610, p < 0.001), and work engagement was also related to resilience (r = 0.632, p < 0.001). In the Saudi sample, the correlations were still moderate but worthy of observation, as mindfulness was correlated to work engagement (r = 0.333, p = 0.006), mindfulness was associated with resilience (r = 0.458, p < 0.001), and work engagement was related to resilience as well (r = 0.298, p = 0.011). The results show a combined effectiveness of the intervention, whereby it promoted each aspect independently but facilitated a stronger connection between them -especially among Egyptian nurses. These findings further suggest that developing mindfulness could be one foundational approach to fostering engagement and resilience within clinical settings.

**Table 7:** Correlation between the complete five-facet mindfulness questionnaire, the utrecht work engagement scale, and the resiliency scale.

	Egypt						Saudi					
	Complete five-facet mindfulness questionnaire		Utrecht work engagement scale		Resiliency scale		Complete five-facet mindfulness questionnaire		Utrecht work engagement scale		Resiliency scale	
	r	p	r	p	r	p	r	p	r	p	r	p
Pre – intervention												
Complete Five-Facet Mindfulness Questionnaire			0.074	0.576	0.011	0.931			0.054	0.681	0.058	0.661
Utrecht Work Engagement Scale	0.074	0.576			0.027	0.837	0.054	0.681			0.093	0.480
Resiliency scale	0.011	0.931	0.027	0.837			0.058	0.661	0.093	0.480		
Post – intervention												
Complete Five-Facet Mindfulness Questionnaire			0.509	<0.001**	0.610	<0.001**			0.333	0.006*	0.458	<0.001**
Utrecht Work Engagement Scale	0.509	<0.001**			0.632	<0.001**	0.333	0.006*			0.298	0.011*
Resiliency scale	0.610	<0.001**	0.632	<0.001**			0.458	<0.001**	0.298	0.011*		

*Statistically significant at p < 0.05.

**Statistically significant at p < 0.01.

**Figure 7:**
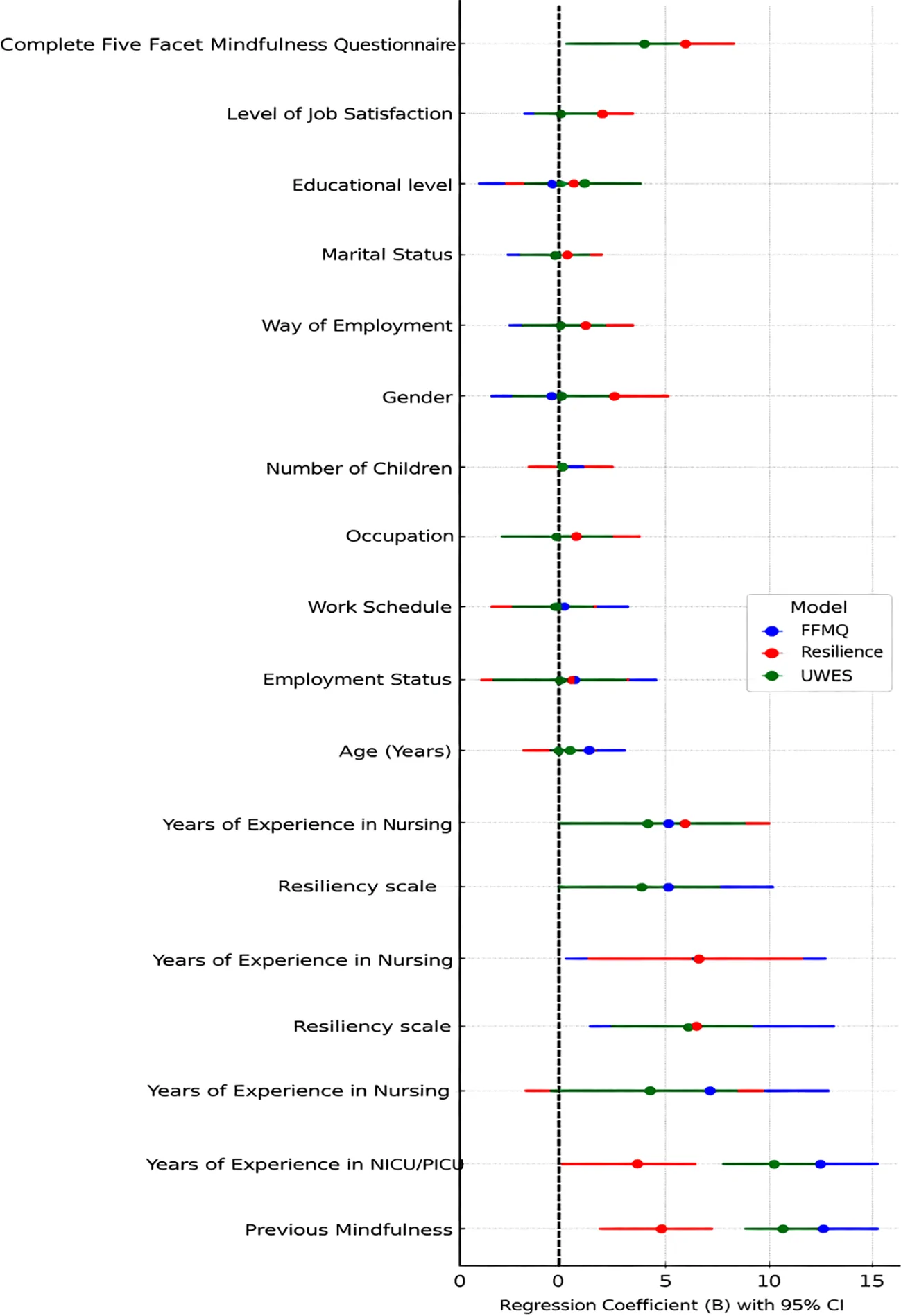
Predictors of mindfulness, resilience, and work engagement among Egyptian nurses.

**Figure 7** summarizes the regression coefficients and 95% confidence intervals for the predictors of Mindfulness (FFMQ), Work Engagement (UWES), and Resilience in Egyptian nurses, providing a visual comparison of how different socio-demographic and psychological variables influence each outcome. The strongest predictors, with higher coefficients and narrower confidence intervals, include Previous Mindfulness, Years of Experience in NICU/PICU, and Resilience Scale, which showed strong positive relationships across all outcomes. Notably, Complete FFMQ scores and Work Engagement emerged as important predictors of Resilience, further illustrating the relationship between these constructs. On the other hand, socio-demographic variables such as gender, marital status, level of education, and number of children had little to no individual predictive impact and were characterized by wider confidence intervals, reflecting limited predictive value. Overall, the story is that experience and psychological factors, specifically previous exposure to mindfulness and clinical experience, are fundamental predictors of well-being, engagement, and resilience, making these compelling areas of intervention and training focus in stressful health care environments.

**Figure 8:**
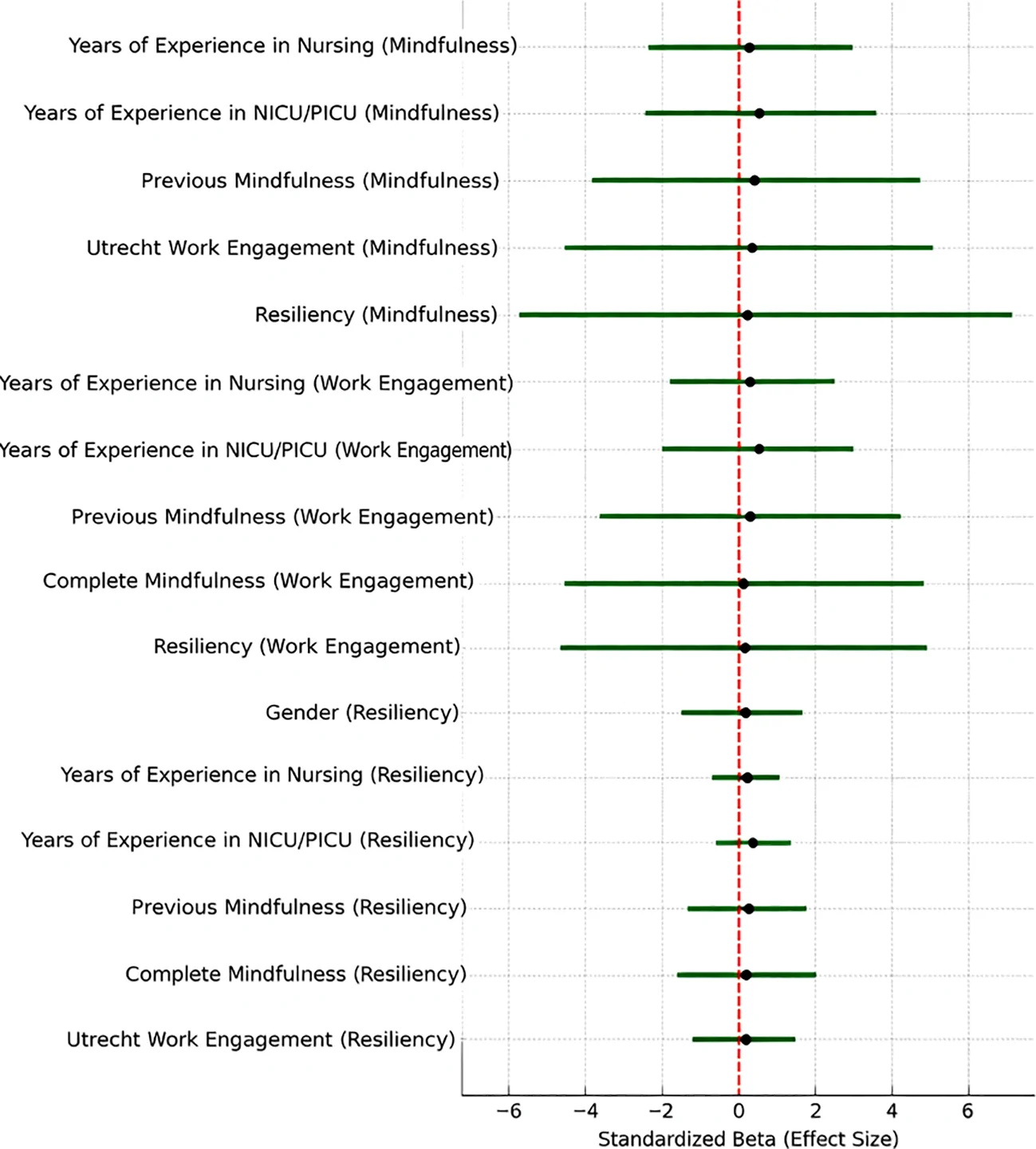
Predictors of mindfulness, work engagement, and resilience among Saudi nurses. Green lines indicate significant predictors (p < 0.05). Gray lines would indicate non-significant predictors (none in this set). Black dots show the standardized Beta for each predictor, and the red dashed line at 0 represents no effect.

The integrated **Figure 8** presents standardized beta coefficients for determinants of Mindfulness, Work Engagement, and Resilience in Saudi nurses and is an appealing visual representation of differences in effect sizes across psychological and experiential variables. The two most pronounced predictors (largest positive effect sizes) were Complete Mindfulness and Work Engagement, identified as two of the larger forces for Resilience, confirming the interdependence of all three variables. Likewise, Resilience and Work Engagement were the most evident predictors of Mindfulness, suggesting a scenario in which psychological resilience and satisfaction in work together enhance mindfulness. Duration of employment time in NICU/PICU, and previous exposure to Mindfulness had moderate positive effects on different outcomes, suggesting that although the clinical experience matters, previous training can also have an influence on psychological well-being. Pertinently, gender and nursing experience in relation to demographics hardly made a difference. Overall, the messaging in the narrative here centers as much on ‘relational’ and ‘experiential’ and psychological aspects, rather than demographic aspects, as being most likely to influence the advancement of mindfulness, engagement, and resilience in this group of clinical practitioners.

## Discussion

### Demographic comparability and workforce context

This study evaluated the effectiveness of structured mindfulness training in enhancing emotional resilience and job engagement among NICU and PICU nurses in Egypt and Saudi Arabia. The demographic similarity between groups—age, gender, education, years of experience, and work schedules—strengthens the internal validity of the study, minimizing baseline disparities as potential confounders. Most nurses were between 25 and 44 years old, with a predominance of women and rotating shift schedules. This aligns with global workforce data showing that NICU nurses are often over 40 years of age, reflecting the specialty’s demand for advanced clinical expertise, emotional maturity, and extensive experience in high-acuity pediatric care (47, 48). The need for specialized certifications and postgraduate training before NICU/PICU roles contributes to this age distribution, while professional stability and high job satisfaction facilitate retention (48).

The educational differences between the two countries highlight variations in workforce structure and health policy. While Saudi nurses were more likely to hold diplomas, Egypt had a slightly larger proportion of nurses with postgraduate degrees than Saudi Arabia. This pattern may stem from Saudi Arabia’s reliance on internationally recruited staff to address workforce shortages, while Egypt’s expanding academic infrastructure has supported growth in advanced nursing education.

### Cultural, organizational, and contextual moderators

Cross-national variations in training outcomes may reflect differences in cultural norms, social support structures, and workplace environments. Prior research indicates that family cohesion and collectivist social support systems—commonly observed in Middle Eastern and North African (MENA) contexts—can serve as protective factors that buffer work-related stress and enhance engagement in psychological interventions (49–51). Organizational dynamics, including leadership support, staffing models, and institutional readiness for wellness initiatives, have also been identified as key determinants of the effectiveness and uptake of mindfulness and resilience programs in healthcare settings (52) Furthermore, spiritual and religious practices such as prayer, contemplation, and reflective rituals—which are deeply embedded in both Saudi and Egyptian cultural life have been shown to complement mindfulness principles and contribute to enhanced emotional regulation and wellbeing (53). These contextual factors collectively provide a theoretically grounded explanation for the observed variations in program outcomes across the two settings. The observed differences between Egyptian and Saudi nurses may be influenced by contextual factors such as cultural norms, organizational structures, and spiritual orientations. However, these interpretations should be regarded as possible explanations rather than definitive conclusions, as the present study did not collect empirical data directly measuring these dimensions. Future research is needed to systematically examine how cultural and organizational contexts contribute to variations in nursing practice across different settings.

### Mechanisms of change and theoretical integration

The mindfulness intervention improved all five core mindfulness dimensions: observing, describing, acting with awareness, non-judging, and non-reactivity, suggesting enhanced attentional control, emotion regulation, and self-compassion. These findings are consistent with the Stress Appraisal Theory, which posits that mindfulness reframes stress responses, enabling effective coping in high-pressure environments such as ICUs (54).

The Job Demands–Resources (JD-R) model provides a strong theoretical framework for understanding these outcomes. Mindfulness acts as a personal resource that mitigates job strain, reduces burnout, and fosters engagement when combined with organizational support such as teamwork and leadership (55). This explains why resilience improved rapidly after training, whereas engagement gains were more modest, as engagement often requires sustained personal and organizational resources to consolidate.

### Comparative evidence and literature alignment

Our findings are consistent with those of prior studies demonstrating that mindfulness-based interventions (MBIs) reduce burnout, improve resilience, and enhance engagement in high-acuity nursing environments (56). Evidence from PICU nurses similarly supports improvements in vigor, dedication, and absorption following brief MBIs (57), while systematic reviews have confirmed that structured MBIs integrated into workflows are especially effective (58).

However, variability across studies highlights the importance of cultural tailoring, delivery format, and training intensity. For example, Elkady (59) reported only modest reductions in burnout, whereas Abdalrahim et al. (60) found significant decreases in emotional exhaustion in structured programs. Brief or app-based interventions generally yield smaller effect sizes than instructor-led programs (61). These findings emphasize the value of personalized and context-specific mindfulness training approaches.

### Resilience and engagement outcomes

This study demonstrated significant post-intervention improvements in both resilience and engagement. Egyptian nurses showed higher rates of “high engagement” than Saudi nurses, potentially reflecting stronger social support systems and differences in workplace demands. Resilience gains are particularly noteworthy given their association with reduced burnout and turnover and their role in sustaining engagement over time (62).

These results are clinically meaningful in the context of persistent psychological distress and burnout among critical care nurses worldwide (63). Enhancing resilience through mindfulness training not only improves staff well-being but also positively affects patient care quality and workforce retention.

### Post-intervention correlates and predictors

Years of NICU/PICU experience emerged as a strong predictor of resilience post-training, supporting the evidence that resilience in high-stress specialties is largely skill- and experience-based rather than innate (64). Prior exposure to mindfulness further amplifies training benefits, reinforcing a dose–response relationship (65).

Mindfulness, resilience, and engagement became strongly interrelated after the intervention. This interconnectedness aligns with JD-R pathways, where mindfulness strengthens attentional control and emotional stability, facilitating resilience and ultimately supporting engagement (66). A positive feedback loop also emerged: nurses with higher baseline engagement and resilience experienced greater mindfulness improvements, echoing evidence that greater motivation and active participation enhance training outcomes (67).

### Practice implications

These findings have several practical implications for healthcare organizations and nursing leadership. To sustain improvements in engagement, MBIs should be paired with structural enhancements consistent with the JD-R model, such as protected micro-breaks, peer-led practice groups, and visible leadership endorsement of mindfulness practice (68). Tailored support is also essential for novice nurses or those unfamiliar with mindfulness, as prior exposure and experience predict better outcomes. Strategies such as short pre-program primers, micro-practices incorporated into handovers, and app-based reminders can foster accessibility and adoption (69).

Future research should use longitudinal designs to examine the sustained effects of MBIs on resilience, engagement, and retention, while exploring their scalability and adaptability across diverse clinical settings.

### Strengths and limitations of the current study

This study has several significant strengths. It stands out as one of the limited quasi-experimental studies that compare mindfulness-based training in two Middle Eastern nations, thereby enhancing its cross-cultural applicability and its external validity. The use of validated tools to evaluate mindfulness, resilience, and work engagement bolstered the reliability of the results. Additionally, this study focused on a particularly vulnerable group, NICU and PICU nurses, who are frequently underrepresented in intervention research despite their high susceptibility to burnout. The intervention was concise and well-structured, proving its feasibility for incorporation into demanding clinical schedules. However, this study has several limitations that should be acknowledged. Its quasi-experimental, non-randomized design without a control group limits causal interpretation and generalizability, and the study was not registered as a clinical trial. Although the protocol was standardized, some participants attended sessions online, which may have reduced engagement and intervention fidelity. The training was delivered by a single certified instructor, which may affect reproducibility. Self-reported measures may introduce bias, and the absence of qualitative data limits a deeper insight into participant experiences. Long-term effects were not assessed, cultural and organizational factors were not fully explored, and the resource-intensive nature of instructor-led training may limit scalability. Future research should use randomized designs, consistent delivery modes, multiple trainers, and extended follow-up.

## Recommendations

1. Incorporating Mindfulness into Ongoing Education: Structured mindfulness-based interventions (MBIs) should be integrated into continuous professional development programs for nurses in intensive care and pediatrics to bolster their coping mechanisms and psychological resilience.
2. Organizational Backing for Mindfulness Initiatives: Hospital management should provide dedicated time, space, and resources for mindfulness training, with a focus on leadership participation to enhance program engagement.
3. Programs should integrate local cultural traditions, religious beliefs, and existing wellness initiatives to enhance accessibility and sustainability.
4. Incorporating the Hybrid Delivery Approaches in providing mindfulness through a combination of in-person workshops and digital tools can enhance scalability and accommodate shift work schedules.
5. Ongoing Evaluation and Follow-Up: Long-term assessments should track the enduring impact of MBIs on resilience, engagement, burnout, and staff retention to improve the interventions over time.

Nursing leaders and health policymakers should incorporate mindfulness-based training into broader workforce well-being strategies to promote safer patient care and increased nursing satisfaction.

## Recommendations for future research

- Mixed-methods studies capturing both quantitative outcomes and qualitative experiences of nurses participating in mindfulness interventions (70).
- Longitudinal follow-up to evaluate the sustainability of the effects of mindfulness on resilience and engagement (71).
- Objective physiological measures, such as heart rate variability or cortisol levels, can complement self-reported data and reduce bias (72).
- Cross-cultural adaptation studies are needed to determine the most effective intervention strategies for diverse nursing populations (73).
- Integration into nursing curricula for early career nurses, particularly in critical care specialties, to build resilience from the beginning of professional practice (74).

## Practical implications for nursing practice

- Mindfulness interventions can be incorporated into routine professional development programs to enhance nurses’ emotional resilience, engagement, and coping skills (75).
- Hospitals should implement scalable delivery models, including mobile applications, blended learning, or brief guided mindfulness sessions, to accommodate shift work and reduce resource demand (76).
- Organizational support, such as wellness policies, mentorship programs, and peer support groups, can enhance the sustainability of mindfulness benefits (53).
- Emphasizing emotional self-care and stress management within orientation and onboarding programs may prepare early-career ICU nurses to thrive in high-acuity environments (77).

## Conclusion

This study demonstrates that structured mindfulness training is a practical, effective, and scalable intervention for strengthening emotional resilience and engagement among NICU and PICU nurses. By enhancing emotion regulation, coping skills, and attentional control, mindfulness training addresses critical workforce challenges, including burnout, psychological distress, and turnover, particularly in high-acuity pediatrics settings. The findings emphasize the value of integrating mindfulness into routine professional development, supported by organizational policies and leadership commitments. Future research should explore the long-term sustainability of mindfulness-based interventions, their impact on patient outcomes, and their adaptability to diverse cultural and healthcare contexts.

## Data Availability

The original contributions presented in the study are included in the article/**Supplementary Material**. Further inquiries can be directed to the corresponding author.
