# Correction: The impact of mindfulness training on emotional resilience and job engagement among NICU and PICU Saudi and Egyptian nurses: a quasi experimental comparative study

**DOI:** 10.3389/fpsyt.2026.2023244

**Published:** 2026-10-27

**Authors:** Amal I. Khalil, Abeer Esawi, Ebtesam Abdulshakoor, Omayma M. Abu Samra

**Affiliations:** 1 King Abdullah International Medical Research Center, Jeddah, Saudi Arabia; 2 College of Nursing, King Saud bin Abdulaziz University for Health Sciences, Jeddah, Saudi Arabia; 3 Ministry of National Guard Health Affairs (MNGHA), Jeddah, Saudi Arabia; 4 Faculty of Nursing, Menoufia University, Shebeen El-Kom, Egypt; 5 Faculty of Nursing, Cairo University, Cairo, Egypt; 6 Faculty of Nursing, Mansoura University, Mansoura, Egypt

**Keywords:** mindfulness, emotional resilience, job engagement, NICU, PICU, nurses, Saudi Arabia, Egypt

The **Reference** for Pehlivan et al. (2022) was erroneously written as “Prendergast AE, Jim KK, Marnas H, Desban L, Quan FB, Djenoune L, et al. CSF-contacting neurons respond to Streptococcus pneumoniae and promote host survival. Curr Biol. (2023) 33:940–956.e10. doi:10.1016/j.cub.2023.01.025”. It should be “Pehlivan T, Semerci R, Çalışkan B, Yıldız G, Altınel N, Kapanci M. The effect of a mindfulness-based stress reduction program on the pediatric hematology-oncology nurses’ professional quality of life and perceived stress level: CN38. Ann Oncol. (2022) 33. doi:10.1016/j.annonc.2022.07.363”.

The **Reference** for Egami and Highfield (2023) was erroneously written as “Highfield MEF, Egami S. The effect of a mindfulness phone application on NICU nurses’ professional quality of life. Adv Neonatal Care. (2023) 23–8. doi:10.1097/ANC.0000000000000990”. It should be “Egami S, Highfield MEF. The effect of a mindfulness phone application on NICU nurses’ professional quality of life. Adv Neonatal Care. (2023) 23(3)–E78. doi:10.1097/ANC.0000000000001064”.

**Reference** 52, “Egami S, Highfield MEF. Mindfulness and spirituality in NICU nursing. Adv Neonatal Care. (2023) 23–8. doi:10.1097/ANC.0000000000000990”, duplicates the Egami and Highfield study retained as the corrected **Reference** 4. **Reference** 52 should be removed, with the relevant in-text citation(s) corrected and subsequent references renumbered as necessary.

The original version of this article has been updated.

