# Supplementary material for: The impact of mindfulness training on emotional resilience and job engagement among NICU and PICU Saudi and Egyptian nurses: a quasi experimental comparative study
Source: Front Psychiatry. 2026 Feb 19;16:1701580. doi: 10.3389/fpsyt.2025.1701580 (PMC12961304; doi:10.3389/fpsyt.2025.1701580)
Supplement: Supplementary file 3 [file DataSheet3.pdf]

NRJ24/066/8

Tools of the study

King Abdullah International Medical Research Center
KAIMRC
APPROVED
IRB
Institutional Review Board

Appendix (1)

Demographic Data:

Age: What is your age?

- Under 25
- 25-34
- 35-44
- 45-54
- 55 and above

Gender: What is your gender?

- Male
- Female

Nationality: What is your nationality?

- Saudi
- Egyptian
- Other (please specify): ___________

Educational Level: What is your highest level of education?

- Diploma in Nursing
- Bachelor's Degree in Nursing
- Master's Degree in Nursing
- Doctorate in Nursing
- Other (please specify): ___________

Years of Experience in Nursing: How many years of experience do you have in nursing?

- Less than 1 year
- 1-3 years
- 4-6 years
- 7-10 years
- More than 10 years

Years of Experience in NICU/PICU: How many years of experience do you have specifically in NICU/PICU units?

- Less than 1 year
- 1-3 years
- 4-6 years
- 7-10 years
- More than 10 years

Job Title/Position: What is your current job title/position?

- Staff Nurse
- Head Nurse
- Nurse Practitioner
- Clinical Nurse Specialist
- Other (please specify): ___________

Employment Status: What is your employment status?

- Full-time
- Part-time
- Contract

Work Schedule: What is your typical work schedule?

- Day shift
- Night shift
- Rotating shifts
- Other (please specify): ____________

Marital Status: What is your marital status?

- Single
- Married
- Divorced
- Widowed

Number of Children: How many children do you have?

- None
- 1
- 2
- 3
- 4 or more

Previous Mindfulness or Stress Management Training: Have you previously participated in any mindfulness or stress management training?

- Yes
- No

Level of Job Satisfaction: How satisfied are you with your current job?

- Very satisfied Satisfied Neutral
- Dissatisfied Very dissatisfied

Way of Employment in NICU/PICU: How were you assigned to the NICU/PICU department?

- Volunteered for the position
- Assigned by management
- Transferred from another department
- Other (please specify): ____________

Appendix (2)

2. Utrecht Work Engagement Scale (UWES)

No.	Statement	0	1	2	3	4	5	6
**I.**	**Vigor:**							
1.	At my work, I feel bursting with energy.							
2.	At my job, I feel strong and vigorous.							
3.	When I get up in the morning, I feel like going to work.							
4.	I can continue working for very long periods at a time.							

5.	At my job, I am very resilient, mentally.							
6.	At my work, I always persevere, even when things do not go well.							
**II.**	**Dedication:**							
7.	I find the work that I do full of meaning and purpose.							
8.	I am enthusiastic about my job.							
9.	My job inspires me.							
10.	I am proud of the work that I do.							
11.	To me, my job is challenging.							
**III.**	**Absorption:**							
12.	Time flies when I'm working.							
13.	When I am working, I forget everything else around me.							
14.	I feel happy when I am working intensely.							
15.	I am immersed in my work.							
16.	I get carried away when I'm working.							
17.	It is difficult to detach myself from my job.							

Appendix (3)

3. CD-RISC-10 Items: Resiliency scale

No.	statements	0	1	2	3	4
1.	I am able to adapt when changes occur.					
2.	I can deal with whatever comes my way.					
3.	I try to see the humorous side of things when I am faced with problems.					
4.	Having to cope with stress can make me stronger.					
5.	I tend to bounce back after illness, injury, or other hardships.					
6.	I believe I can achieve my goals, even if there are obstacles.					
7.	Under pressure, I stay focused and think clearly.					
8.	I am not easily discouraged by failure.					
9.	I think of myself as a strong person when dealing with life's challenges and difficulties.					
10.	I am able to handle unpleasant or painful feelings like sadness, fear, and anger.					

Appendix (4)

4. The complete Five Facet Mindfulness Questionnaire (FFMQ)

	Statements	1	2	3	4	5
I.	Observing					
1.	When I'm walking, I deliberately notice the sensations of my body moving.					
2.	I notice the smells and aromas of things.					
3.	I pay attention to how my emotions affect my thoughts and behavior.					
4.	I notice visual elements in art or nature, such as colors, shapes, textures, or patterns of light and shadow.					
5.	I pay attention to sounds, such as clocks ticking, birds chirping, or cars passing.					
6.	I notice the sensations of my body, such as whether it is tense or relaxed.					
7.	I pay attention to the sensations of water when I'm taking a shower or bath.					
II.	Describing					
8.	I'm good at finding words to describe my feelings.					
9.	I can easily put my beliefs, opinions, and expectations into words.					
10.	I'm good at thinking of words to express my perceptions, such as how things taste, smell, or sound.					
11.	It's hard for me to find the words to describe what I'm thinking (reverse-scored).					
12.	I have trouble thinking of the right words to express how I feel about things (reverse-scored).					
13.	When I have a sensation in my body, it's difficult for me to describe it because I can't find the right words (reverse-scored).					
III.	Acting with Awareness					
14.	When I do things, my mind wanders off and I'm easily distracted (reverse-scored).					
15.	When I'm doing something, I'm only focused on what I'm doing, nothing else.					
16.	I find it difficult to stay focused on what's happening in the present (reverse-scored).					
17.	It seems I am "running on automatic" without much awareness of what I'm doing (reverse-scored).					
18.	I rush through activities without being really attentive to them (reverse-scored).					
19.	I do jobs or tasks automatically, without being aware of what I'm doing (reverse-scored).					
20.	I find myself doing things without paying attention (reverse-					

	scored).					
IV.	Non-judging of Inner Experience					
21.	I criticize myself for having irrational or inappropriate emotions (reverse-scored).					
22.	I tell myself I shouldn't be feeling the way I'm feeling (reverse-scored).					
23.	I believe some of my thoughts are abnormal or bad and I shouldn't think that way (reverse-scored).					
24.	I make judgments about whether my thoughts are good or bad (reverse-scored).					
25.	I tell myself that I shouldn't be thinking the way I'm thinking (reverse-scored).					
26.	I think some of my emotions are bad or inappropriate and I shouldn't feel them (reverse-scored).					
V.	Non-reactivity to Inner Experience					
27.	When I have distressing thoughts or images, I just notice them and let them go.					
28.	When I have distressing thoughts or images, I am able just to notice them without reacting.					
29.	When I have distressing thoughts or images, I do not let myself get carried away by them.					
30.	Usually when I have distressing thoughts or images, I can just notice them without reacting.					

King Abdullah International Medical Research Center
KAIMRC
APPROVED
IRB
Institutional Review Board
